# microRNA-199a-3p inhibits hepatic apoptosis and hepatocarcinogenesis by targeting PDCD4

**DOI:** 10.1038/s41389-020-00282-y

**Published:** 2020-10-24

**Authors:** Zhenyang Li, Ye Zhou, Liyuan Zhang, Kaiwei Jia, Suyuan Wang, Mu Wang, Nan Li, Yizhi Yu, Xuetao Cao, Jin Hou

**Affiliations:** National Key Laboratory of Medical Immunology & Institute of Immunology, Second Military Medical University, 200433 Shanghai, China

**Keywords:** Liver cancer, Apoptosis

## Abstract

Hepatic apoptosis and the initiated liver inflammation play the initial roles in inflammation-induced hepatocarcinogenesis. Molecular mechanisms underlying the regulation of hepatocyte apoptosis and their roles in hepatocarcinogenesis have attracted much attention. A set of microRNAs (miRNAs) have been determined to be dysregulated in hepatocellular carcinoma (HCC) and participated in cancer progression, however, the roles of these dysregulated miRNAs in carcinogenesis are still poorly understood. We previously analyzed the dysregulated miRNAs in HCC using high-throughput sequencing, and found that miR-199a/b-3p was abundantly expressed in human normal liver while markedly decreased in HCC, which promotes HCC progression. Whether miR-199a/b-3p participates in HCC carcinogenesis is still unknown up to now. Hence, we focused on the role and mechanism of miR-199a/b-3p in hepatocarcinogenesis in this study. Hepatic miR-199a/b-3p was determined to be expressed by *miR*-*199a*-*2* gene in mice, and we constructed *miR*-*199a*-*2* knockout and hepatocyte-specific *miR*-*199a*-*2* knockout mice. Diethylnitrosamine (DEN)-induced hepatocarcinogenesis were markedly increased by hepatocyte-specific miR-199a-3p knockout, which is mediated by the enhanced hepatocyte apoptosis and hepatic injury by DEN administration. In acetaminophen (APAP)-induced acute hepatic injury model, hepatocyte-specific miR-199a-3p knockout also aggravated hepatic apoptosis. By proteomic screening and reporter gene validation, we identified and verified that hepatic programed cell death 4 (PDCD4), which promotes apoptosis, was directly targeted by miR-199a-3p. Furthermore, we confirmed that miR-199a-3p-suppressed hepatocyte apoptosis and hepatic injury by targeting and suppressing PDCD4. Thus, hepatic miR-199a-3p inhibits hepatocyte apoptosis and hepatocarcinogenesis, and decreased miR-199a-3p in hepatocytes may aggravate hepatic injury and HCC development.

## Introduction

Hepatocellular carcinoma (HCC) is a kind of lethal malignant primary liver cancer and ranks fourth on the cancer-related death list and sixth in the terms of incident cases worldwide, which has a low 5-year survival rate^1,2^. Liver diseases, such as chronic hepatitis caused by hepatitis B or C virus (HBV or HCV), alcoholic liver disease and non-alcoholic fatty liver disease, increase the risk of hepatocellular carcinoma development^3^. Generally, HCC carcinogenesis is caused by the repeated cycle of hepatic injury, induced liver inflammation, and compensatory hepatocyte proliferation^4^. The liver injury plays a critical role in this vicious cycle, and death of hepatocytes is able to release damage-associated molecular patterns (DAMPs), which activates immune cells in the liver, recruits circulatory inflammatory cells, and initiates liver inflammation. The induced liver inflammation stimulates compensatory hepatocyte proliferation, while hepatic inflammatory responses also worsen hepatic damage and aggravate liver inflammation. Sustained hepatic injury induces the chronic liver inflammation and repeated compensatory hepatocyte proliferation, which eventually lead to hepatocarcinogenesis^5,6^. The underlying regulatory mechanisms of hepatocarcinogenesis have attracted much attention but remain largely unknown, which needs further investigation.

Cell death plays pivotal roles in the initiation of hepatocarcinogenesis. Apoptosis, necroptosis, pyroptosis, and ferroptosis are the intensively explored types of cell death during tissue damage, and different types of hepatocyte death eventually lead to different types of liver cancer^7^. For instance, the necroptosis of hepatocytes incubates an environment, which determines intrahepatic cholangiocarcinoma (ICC) outgrowth. However, if hepatocytes are in the environment created by apoptotic hepatocytes, they are inclined to become HCC^8^. Hepatocyte-specific deletion of the IκB kinase (IKK) subunit NEMO/IKKγ sensitizes hepatocyte apoptosis by NF-κB inhibition, which spontaneously forms mouse HCC in 12 months^9^. Similarly, mice lacking the anti-apoptotic myeloid cell leukemia-1 (Mcl-1) in hepatocytes have severe liver damage caused by spontaneous apoptosis, and tumor formation is observed in over 50% of mice in 8 months^10^. Besides, mice with combined knockout of receptor-interacting serine/threonine-protein kinase 1 (RIPK1) and Rel-like domain-containing protein A (RelA) in hepatocytes show increased hepatocyte apoptosis and develop spontaneous HCC^11^. Furthermore, other proteins regulating hepatocyte apoptosis, such as TGF-β-activated kinase 1(TAK1), TNF receptor-associated factor 2 (TRAF2), and IκB kinase subunit beta (IKKβ), also participate in hepatocarcinogenesis^12,13^. Therefore, hepatocyte apoptosis is critical in the initiation of hepatocarcinogenesis, and the molecular regulatory mechanisms of hepatocyte apoptosis and their roles in hepatocarcinogenesis have raised widespread concerns.

MicroRNAs (miRNAs) are a class of single-strand RNA with approximately 22 nucleotides in length. The functions of miRNAs have been widely investigated, and they are determined to play critical roles in cancer progression, especially in HCC. A set of miRNAs, such as miR-330-5p, miR-520a, and miR-483-3p, have been suggested to participate in cancer progression, by modulating proliferation, apoptosis, migration, and invasion^14–16^. However, the roles of miRNAs in hepatocarcinogenesis, especially determined in vivo, are still poorly investigated. Mice with miR-122 knockout spontaneously develop steatohepatitis, fibrosis, and eventually HCC in 11 months^17^. Yet, it remains unknown whether miRNAs can regulate hepatocyte apoptosis and the related hepatocarcinogenesis in vivo up to now.

Previously, we carried out an in-depth analysis of the miRNomes in human normal liver tissues and HCC tissues, and found that the liver-enriched miRNA miR-199a/b-3p was dramatically decreased in HCC, which promotes HCC progression both in vitro and in vivo^18^. However, the potential roles of miR-199a/b-3p in hepatocarcinogenesis is still unknown. Hence, we intend to construct hepatocyte-specific miR-199a/b-3p knockout mice, and explore the role and underlying mechanism of miR-199a/b-3p in hepatocarcinogenesis in this study.

## Results

### Construction of miR-199a-3p knockout mice

**Fig. 1:**
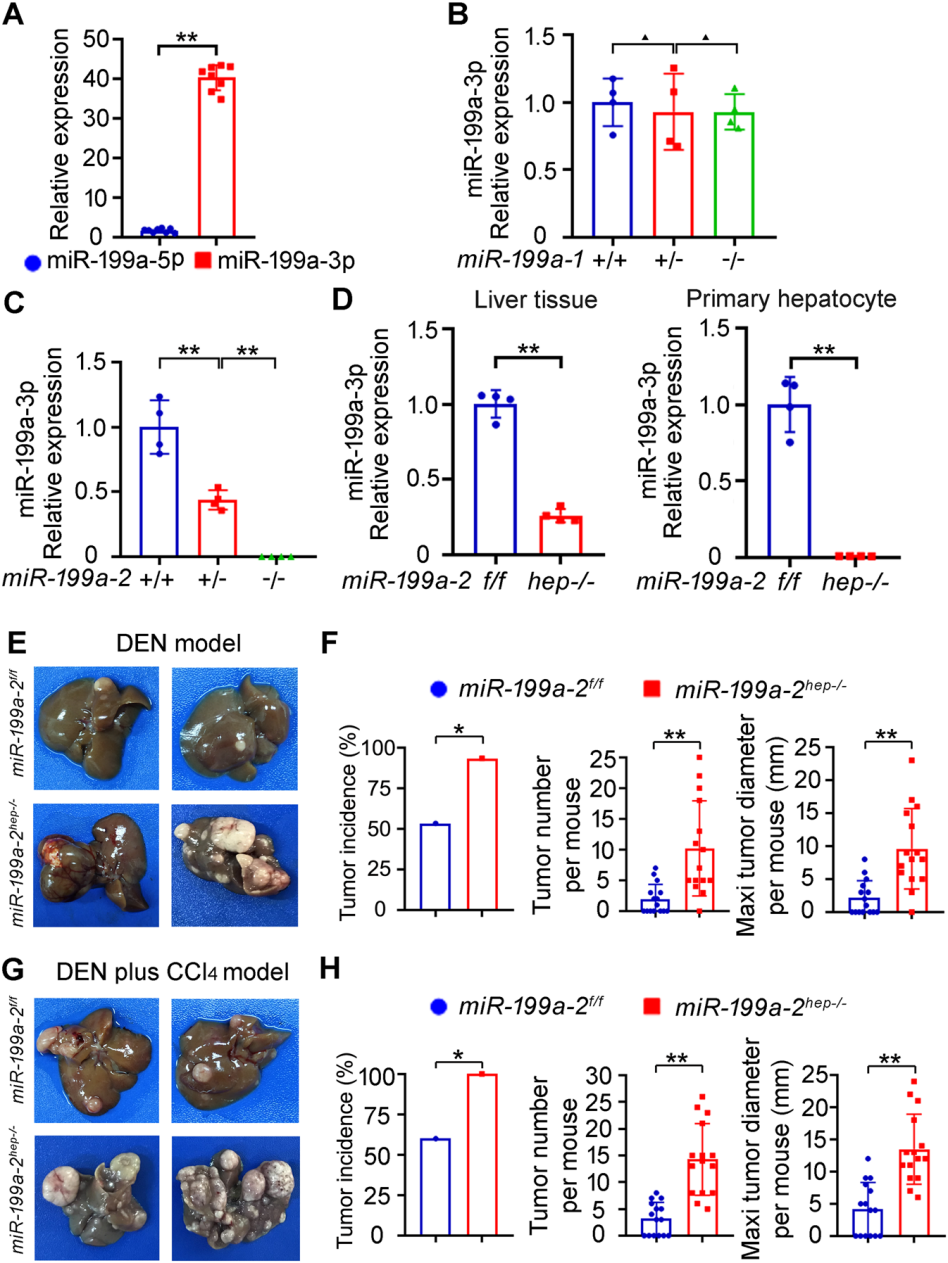
Hepatocyte-specific *miR*-*199a*-*2* knockout promotes hepatocarcinogenesis. **a** The expression of miR-199a-5p and miR-199a-3p was detected by qRT-PCR in mouse liver tissues (*n* = 8). **b** The expression of miR-199a-3p was detected by qRT-PCR from liver tissues of *miR*-*199a*-*1*^+/+^, *miR*-*199a*-*1*^+/-^ and *miR*-*199a*-*1*^-/-^ mice (*n* = 4). **c** The expression of miR-199a-3p was detected by qRT-PCR from liver tissues of *miR*-*199a*-*2*^+/+^, *miR*-*199a*-*2*^+/-^ and *miR*-*199a*-*2*^-/-^ mice (*n* = 4). **d** The expression of miR-199a-3p was detected by qRT-PCR from liver tissues and primary hepatocytes of *miR*-*199a*-*2*^*f/f*^ and *miR*-*199a*-*2*^*hep-/-*^ mice (*n* = 4). **e** Two-week-old *miR*-*199a*-*2*^*f/f*^ and *miR*-*199a*-*2*^*hep-/-*^ male mice were intraperitoneally injected with a single dose of DEN (25 mg/kg) and sacrificed 8 months later. The livers were dissected and photographed. **f** Tumor incidence (chi-square test), tumor number per mouse (unpaired *t*-test), and maximal tumor diameter per mouse (unpaired *t*-test) of the DEN-induced HCC in *miR*-*199a*-*2*^*f/f*^ and *miR*-*199a*-*2*^*hep-/-*^ mice were shown, respectively, as indicated (*n* = 15). **g** Two-week-old *miR*-*199a*-*2*^*f/f*^ and *miR*-*199a*-*2*^*hep-/-*^ male mice were intraperitoneally injected with DEN and then CCl_4_. The livers with DEN plus CCl_4_-induced HCC were dissected and photographed. **h** Tumor incidence (chi-square test), tumor number per mouse (unpaired *t-*test), and maximal tumor diameter per mouse (unpaired *t*-test) of the DEN plus CCl_4_-induced HCC in *miR*-*199a*-*2*^*f/f*^ and *miR*-*199a*-*2*^*hep-/-*^ mice were shown, respectively, as indicated (*n* = 15). Data are shown as mean ± SD or typical photographs of one representative experiment. Similar results were obtained in three independent experiments. ^▲^*P* > 0.05; *P < 0.05; **P < 0.01.

We previously analyzed the dysregulated miRNAs in HCC using high-throughput sequencing, and found that miR-199a/b-3p was abundantly expressed in human normal liver while markedly decreased in HCC, which promotes HCC progression^18^. In order to elucidate the roles of miR-199a/b-3p in hepatocarcinogenesis, we intended to knock out its expression in mouse liver. As miR-199a/b-3p is expressed mainly by *miR*-*199a*-*2* gene (Chr 1) in human liver, we first examined the expression of *miR-199a* gene in mouse liver. miR-199a-3p was found to be abundantly expressed in mouse liver while the miR-199a-5p strand was nearly not expressed (Fig. 1a), which is similar to those in human liver. There are two gene loci of *miR-199a*, *miR*-*199a*-*1* and *miR*-*199a*-*2*, which may both express miR-199a-3p. We then constructed the *miR*-*199a*-*1* or *miR*-*199a*-*2* conventional knockout mice, respectively, to determine which one is mature miR-199a-3p primarily from (Supplementary Fig. S1A, B). These generated knockout mice are fertile and seem normal. Among them, mature miR-199a-3p expression was not influenced by knockout of *miR*-*199a*-*1* gene (Fig. 1b), while knockout of *miR*-*199a*-*2* gene deleted the expression of miR-199a-3p in the liver (Fig. 1c). Therefore, we determined that the mature miR-199a-3p was mainly expressed from *miR*-*199a*-*2* gene in mouse liver.

In order to investigate the role of miR-199a-3p exclusively in hepatocytes and hepatocarcinogenesis, we then constructed the *miR*-*199a*-*2* floxed mice and crossed them with Alb-cre mice to generate hepatocyte-specific *miR*-*199a*-*2* knockout mice (Supplementary Fig. S1, C). There is no obvious difference in body weight and size between *miR-199a-2*^*f/f*^ and *miR-199a-2*^*hep-/-*^ mice, and they are both fertile. In 24-week-old *miR-199a-2*^*f/f*^ and *miR-199a-2*^*hep-/-*^ mice, serum ALT, AST, and GGT levels are similar, suggesting no spontaneous liver injury by hepatic *miR-199a-2* knockout (Supplementary Fig. S1D). The livers of *miR-199a-2*^*f/f*^ and *miR-199a-2*^*hep-/-*^ mice were examined by HE staining, and no abnormal hepatic structure was found in *miR-199a-2*^*hep-/-*^ mice (Supplementary Fig. S1E). Altogether, no obvious spontaneous hepatic disease is induced in *miR-199a-2*^*hep-/-*^ mice. In *miR-199a-2*^*hep-/-*^ mice, miR-199a-3p expression was markedly decreased in the liver tissue, while abolished in the isolated hepatocytes (Fig. 1d). Thus, we constructed the genetic mouse model of hepatic miR-199a-3p knockout, so as to investigate the role of miR-199a-3p in hepatocarcinogenesis.

### Hepatocyte-specific *miR*-*199a*-*2* knockout promotes hepatocarcinogenesis

To study the role of miR-199a-3p in hepatocarcinogenesis, the DEN-induced hepatocarcinogenesis model and DEN plus CCl_4_ model were applied (Supplementary Fig. S1F)^19,20^. In the DEN model, mice were intraperitoneally injected with DEN 14 days post birth, and were sacrificed after 8 months. In contrast to *miR*-*199a*-*2*^*f/f*^ mice, the induced hepatocarcinogenesis was markedly enhanced in *miR*-*199a*-*2*^*hep-/-*^ mice (Fig. 1e), including increased tumor incidence, number, and maximal diameter (Fig. 1f). Similarly, in the DEN plus CCl_4_ model, hepatocyte-specific miR-199a-3p knockout also markedly promoted hepatocarcinogenesis, including the increased tumor incidence, number, and maximal diameter (Fig. 1g, h). Hence, we concluded that hepatocyte-specific miR-199a-3p knockout remarkably promoted hepatocarcinogenesis in vivo.

### Hepatic *miR*-*199a*-*2* knockout promotes hepatocyte apoptosis and hepatic injury

**Fig. 2:**
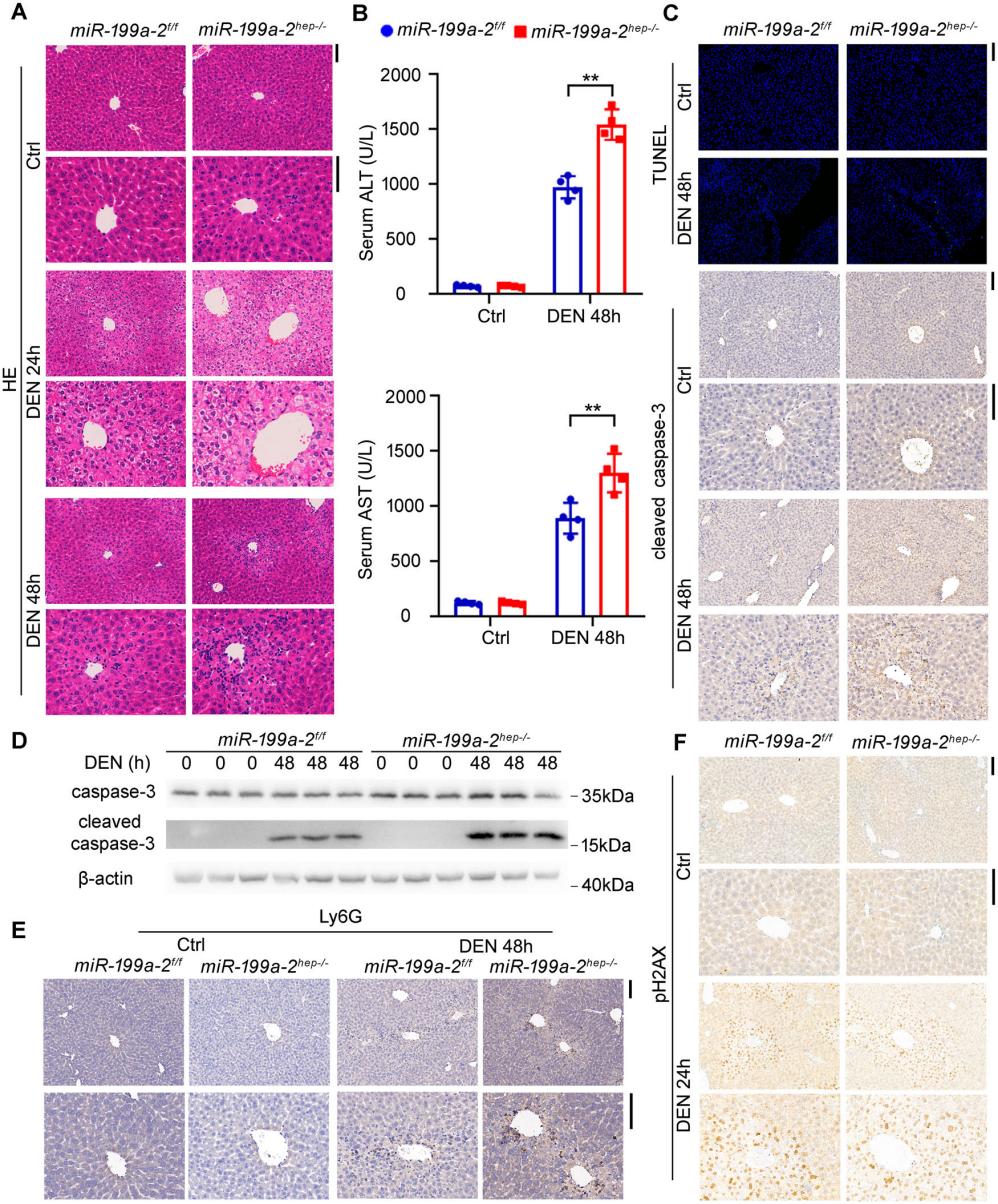
Hepatocyte-specific miR-199a-3p knockout promotes DEN-induced hepatocyte apoptosis and hepatic injury. Eight-week-old *miR*-*199a*-*2*^*f/f*^ and *miR*-*199a*-*2*^*hep-/-*^ male mice were intraperitoneally injected with DEN (100 mg/kg). Livers and serum were collected at the indicated time points. HE staining of liver sections (**a**), Serum ALT and ALT (*n* = 4) (**b**), TUNEL and cleaved caspase-3 IHC staining (**c**), cleaved caspase-3 blot (**d**), Ly6G staining for inflammatory cell infiltration (**e**), and pH2AX staining for DNA damage (**f**) were measured in the indicated time points post DEN challenge. Data are shown as mean ± SD or typical photographs of one representative experiment. Similar results were obtained in three independent experiments. **P < 0.01. Scale bars, 100 μm.

To illustrate the corresponding mechanism responsible for hepatic miR-199a-3p knockout-promoted hepatocarcinogenesis, *miR*-*199a*-*2*^*f/f*^ and *miR*-*199a*-*2*^*hep-/-*^ mice were injected with high-dose DEN to induce acute hepatic injury and liver inflammation. After DEN challenge, ballooning degeneration and coagulative necrosis were observed in the peri-central hepatocytes, which were more severe in *miR*-*199a*-*2*^*hep-/-*^ mice than those in *miR*-*199a*-*2*^*f/f*^ mice (Fig. 2a). Besides, the level of serum ALT and AST post DEN injection in *miR*-*199a*-*2*^*hep-/-*^ mice were also significantly higher than those in *miR*-*199a*-*2*^*f/f*^ mice (Fig. 2b). Correspondingly, TUNEL staining and cleaved caspase-3 IHC staining determined that hepatocyte apoptosis in *miR*-*199a*-*2*^*hep-/-*^ mice was significantly more severe than that in *miR*-*199a*-*2*^*f/f*^ mice, which was confirmed by the increased cleaved caspase-3 in the liver of *miR*-*199a*-*2*^*hep-/-*^ mice (Fig. 2c, d). These data suggest that hepatocyte-specific miR-199a-3p knockout promoted the hepatocyte apoptosis and hepatic injury following DEN injection.

The infiltration of inflammatory cells and compensatory proliferation of hepatocytes following acute DEN injection were also examined in the liver of *miR*-*199a*-*2*^*hep-/-*^ mice, and they were only slightly increased as compared to those in *miR*-*199a*-*2*^*f/f*^ mice (Fig. 2e and Supplementary Fig. S2A). Furthermore, the damage of DEN, determined by the hepatocyte DNA damage, showed no significant difference in the livers between *miR*-*199a*-*2*^*f/f*^ and *miR*-*199a*-*2*^*hep-/-*^ mice (Fig. 2f). Thus, the increased hepatic injury and hepatocyte apoptosis in the liver of *miR*-*199a*-*2*^*hep-/-*^ mice may be mediated by the direct enhancement of apoptosis under miR-199a-3p knockout.

**Fig. 3:**
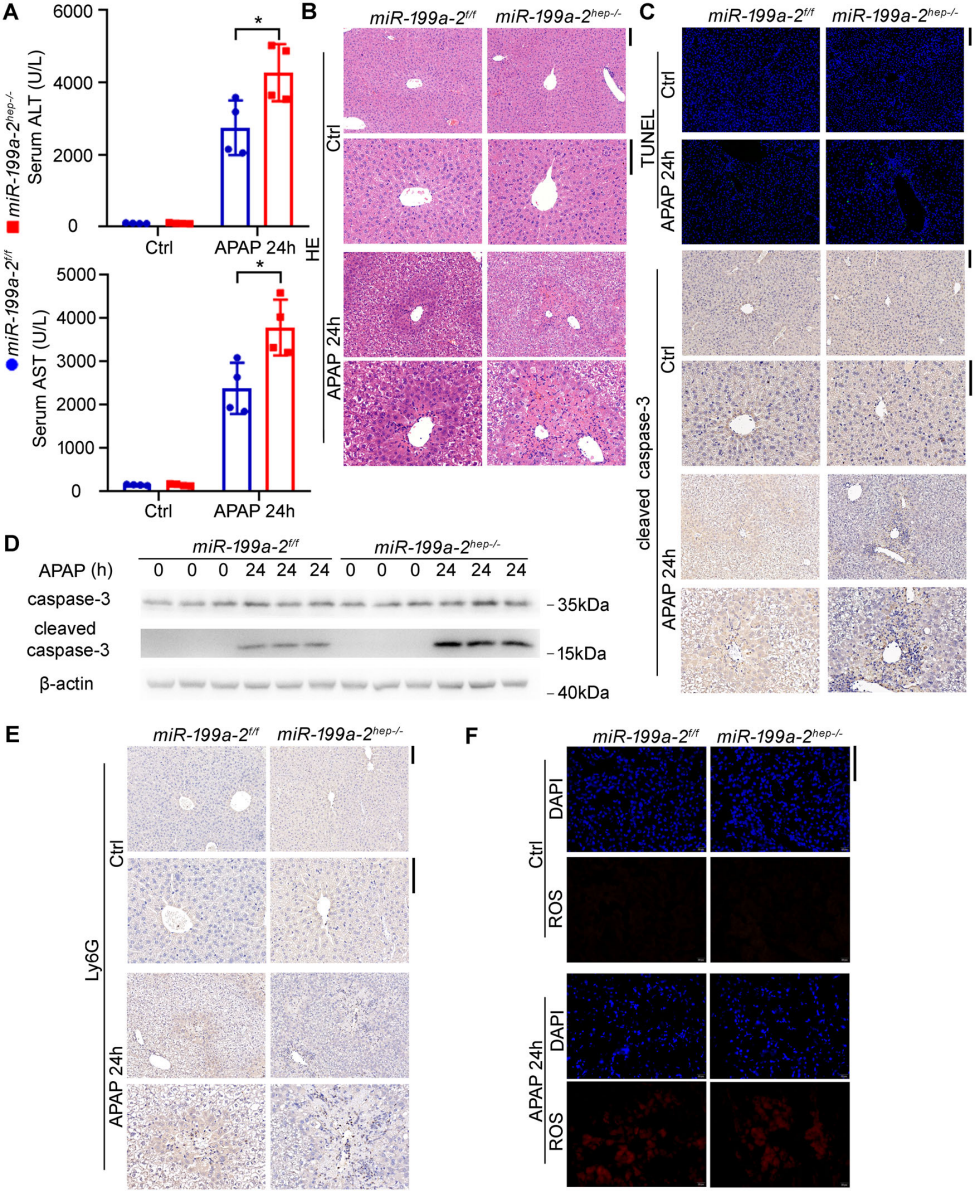
Hepatocyte-specific miR-199a-3p knockout promotes APAP-induced hepatocyte apoptosis and hepatic injury. Eight-week-old *miR*-*199a*-*2*^*f/f*^ and *miR*-*199a*-*2*^*hep-/-*^ male mice were fasted for 16 h and then intraperitoneally injected with APAP (400 mg/kg). Livers and serum were obtained at the indicated time points. Serum ALT and ALT (*n* = 4) (**a**), HE staining (**b**), TUNEL and cleaved caspase-3 IHC staining (**c**), cleaved caspase-3 blot (**d**), Ly6G staining for inflammatory cell infiltration (**e**), and DHE staining for ROS (**f**) were measured in the indicated time points post APAP injection. Data are shown as mean ± SD or typical photographs of one representative experiment. Similar results were obtained in three independent experiments. *P < 0.05. Scale bars, 100 μm.

To further determine the role of miR-199a-3p in hepatocyte apoptosis and hepatic injury, we applied the hepatic injury mouse model using acetaminophen (APAP), an anti-pyretic drug causing liver injury or even hepatic failure by misuse^21^. In the APAP-challenged *miR*-*199a*-*2*^*f/f*^ and *miR*-*199a*-*2*^*hep-/-*^ mice, serum ALT and AST indicating liver injury were significantly increased by hepatocyte-specific miR-199a-3p knockout (Fig. 3a). Hepatic injury around the central vein were also much heavier in the liver of in *miR*-*199a*-*2*^*hep-/-*^ mice than that in *miR*-*199a*-*2*^*f/f*^ mice (Fig. 3b). Besides, TUNEL and cleaved caspase-3 staining in the liver sections showed significantly more apoptotic peri-central hepatocytes in *miR*-*199a*-*2*^*hep-/-*^ mice, which was confirmed by western blot (Fig. 3c, d). The infiltrated inflammatory cells were increased in the damaged hepatic region of *miR*-*199a*-*2*^*hep-/-*^ mice, and the compensatory hepatocyte proliferation was also increased (Fig. 3e and Supplementary Fig. S2B). Additionally, the direct damage of APAP, which is the induced reactive oxygen species (ROS) in hepatocytes, was similar in the liver between *miR*-*199a*-*2*^*f/f*^ and *miR*-*199a*-*2*^*hep-/-*^ mice (Fig. 3f). Together, all these data suggest that hepatocyte apoptosis were enhanced by miR-199a-3p knockout, which may be responsible for the increased hepatocarcinogenesis.

### Hepatic miR-199a-3p knockout promotes apoptosis in vitro

**Fig. 4:**
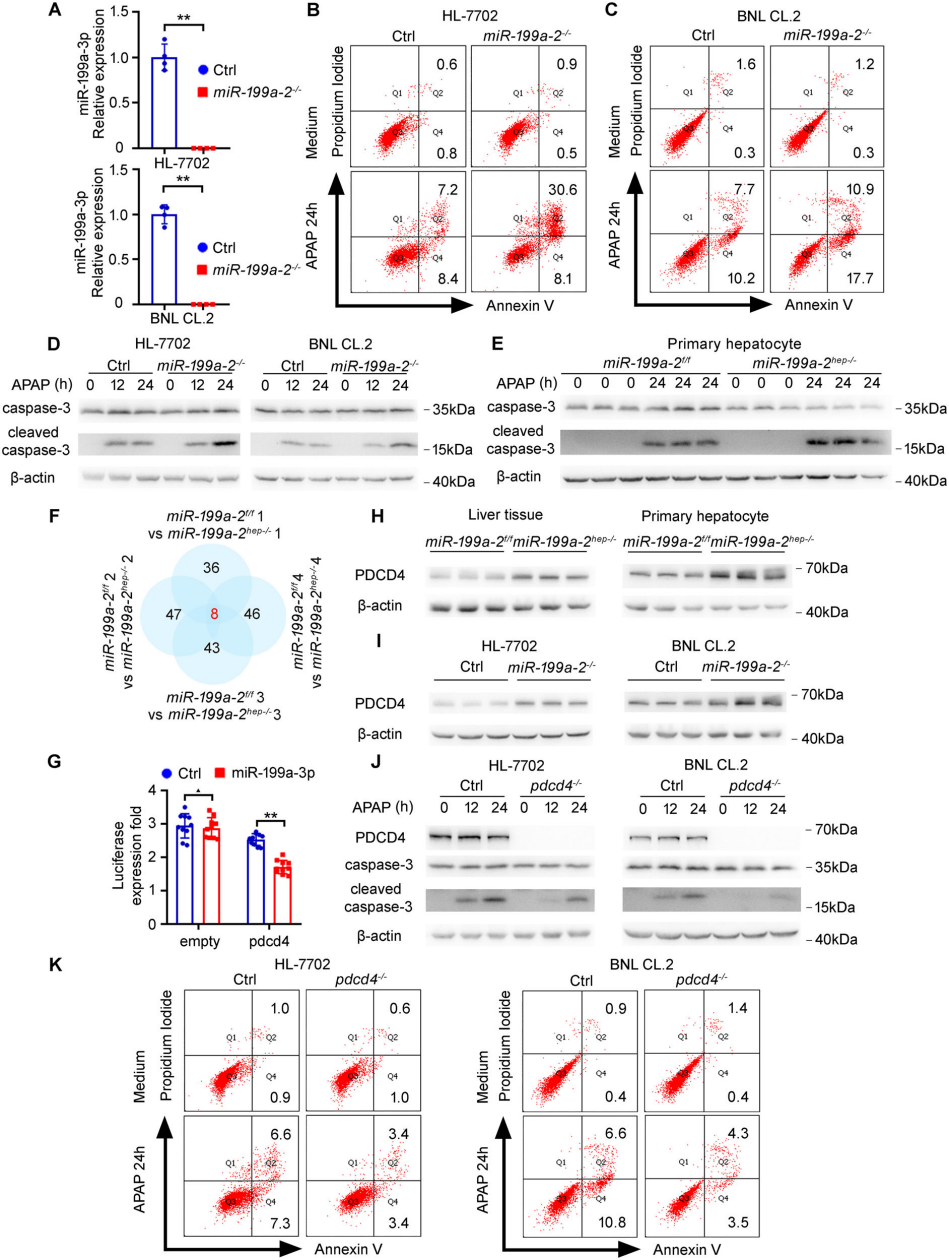
Loss of miR-199a-3p promotes hepatocyte apoptosis by directly targeting pro-apoptotic PDCD4. **a** The expression of miR-199a-3p was detected by qRT-PCR in control and *miR-199a-2*^*-/-*^ cell lines. **b**–**e** Control or *miR*-*199a*-*2* knockout hepatocyte cell lines were treated by APAP (10 mM) for 24 h as indicated. Cell apoptosis was analyzed by flow cytometry (**b**, **c**), and cleaved caspase-3 was examined by Western blot (**d**, **e**). **f** The comparative differential proteins in liver tissues from *miR*-*199a*-*2*^*f/f*^ and *miR-199a*-*2*^*hep-/-*^ mice were shown as indicated, which were from four biological repeats. **g** The indicated Firefly luciferase reporter plasmids and miR-199a-3p or control mimics were transfected into the HEK 293 T cells. After 24 h, Firefly luciferase activity was measured and normalized by Renilla luciferase activity (*n* = 10). **h** Protein level of PDCD4 in liver tissues or primary hepatocytes from *miR*-*199a*-*2*^*f/f*^ and *miR-199a*-*2*^*hep-/-*^ mice was measured by Western blot. **i** Protein level of PDCD4 in control or *miR*-*199a*-*2* knockout hepatocyte cell lines was measured by western blot. **j, k** Control and *pdcd4* knockout hepatocyte cell lines were treated with APAP for the indicated time points. PDCD4, caspase-3, and cleaved caspase-3 were measured by Western blot (**j**), and cell apoptosis was measured by flow cytometry (**k**). Data are shown as mean ± SD or typical photographs of one representative experiment. Similar results were obtained in three independent experiments. ^▲^*P* > 0.05; **P < 0.01.

In order to confirm the pro-apoptotic function of miR-199a-3p knockout in hepatocytes, we used the APAP-induced apoptosis model in vitro, and the *miR*-*199a*-*2* knockout hepatocyte cell lines were constructed and verified (Fig. 4a). As compared to those in control cell lines, *miR*-*199a*-*2* knockout human hepatocyte HL-7702 cells and mouse hepatocyte BNL CL.2 cells exhibited markedly increased apoptosis, respectively, following APAP administration (Fig. 4b, c). The APAP-induced cleaved caspase-3 was also significantly enhanced by miR-199a-3p knockout in these hepatocyte cell lines (Fig. 4d). Moreover, the primary hepatocytes were isolated from *miR*-*199a*-*2*^*f/f*^ and *miR*-*199a*-*2*^*hep-/-*^ mice, respectively, and the APAP-induced cleaved caspase-3 was confirmed to be significantly promoted by *miR*-*199a*-*2* knockout (Fig. 4e). Thus, miR-199a-3p knockout promotes apoptosis in both primary hepatocytes and hepatocyte cell lines in vitro.

### Hepatic miR-199a-3p knockout promotes apoptosis through the mitochondrial pathway

We next intended to figure out the hepatic miR-199a-3p knockout-promoted apoptosis was through death receptor pathway or mitochondrial pathway. The cleavage and activation of caspase-8, characteristic of death receptor apoptosis pathway, were examined in both DEN and APAP-induced hepatocyte apoptosis. Using death receptor pathway activator TNF-α plus CHX in vitro and LPS plus D-gal in vivo as the positive control^22–24^, we did not find the caspase-8 cleavage and death receptor pathway activation in hepatocytes upon DEN or APAP administration (Supplementary Fig. S3A–D). Thus, death receptor apoptosis pathway is not activated in DEN or APAP-induced hepatocyte apoptosis, and miR-199a-3p is less likely to inhibit hepatocyte apoptosis through suppressing death receptor pathway.

In mitochondrial apoptosis pathway, the classical pro-apoptotic BAK and BAX and anti-apoptotic BCL-2 and BCL-X_L_ were examined in DEN or APAP-induced hepatocyte apoptosis. In APAP-induced hepatocyte apoptosis, the levels of these four proteins were not changed both in vitro and in vivo (Supplementary Fig. S3E, F, G). In DEN-induced hepatocyte apoptosis, the levels of both BAX and BCL-X_L_ were increased upon DEN injection (Supplementary Fig. S3H). In *miR-199a-2*^*hep-/-*^ mice, the level of hepatic BCL-X_L_ was significantly decreased as compared to that in *miR-199a-2* ^*f/f*^ mice, and similar results were obtained in hepatocyte cell lines (Supplementary Fig. S3E–H). These data suggest that *miR-199a-2* knockout may inhibit BCL-X_L_ expression, so as to enhance the activation of hepatic mitochondrial apoptosis pathway.

Furthermore, we also examined the release of cytochrome C into the cytosol, which is the characteristic of mitochondrial apoptosis pathway activation. In both DEN and APAP-induced release of cytochrome C, cytosol cytochrome C was significantly increased by hepatic *miR-199a-2* knockout (Supplementary Fig. S3I–L), suggesting that miR-199a-3p knockout promotes the activation of mitochondrial apoptosis pathway in hepatocytes upon DEN or APAP administration.

### Hepatic PDCD4 expression is directly targeted by miR-199a-3p

The corresponding mechanism responsible for miR-199a-3p knockout-promoted apoptosis was then investigated. As miRNAs function mainly through the inhibition of their target genes expression, we screened the differential proteins between the liver tissues from *miR*-*199a*-*2*^*f/f*^ and *miR*-*199a*-*2*^*hep-/-*^ mice using proteomic tandem mass tags (TMT) mass spectrometry (MS) analysis. Among the identified differentially expressed proteins in the liver of *miR*-*199a*-*2*^*f/f*^ and *miR*-*199a*-*2*^*hep-/-*^ mice, eight of them with good reproducibility were chosen for further analysis (Fig. 4f and Supplementary Fig. S4A). By sequence alignment and functional annotation, we focused on the increased PDCD4 in *miR*-*199a*-*2*^*hep-/-*^ liver, which bears miR-199a-3p conserved target sequence and is known for its ability to promote apoptosis (Supplementary Fig. S4B)^25,26^. The direct targeting of PDCD4 mRNA by miR-199a-3p was validated using dual-luciferase reporter assay (Fig. 4g). The mRNA level of PDCD4 was not influenced by miR-199a-3p knockout (Supplementary Fig. S4,C, D), while the protein level of PDCD4 was significantly increased by miR-199a-3p knockout in liver tissues, primary hepatocytes, and hepatocyte cell lines (Fig. 4h, i). Additionally, the pro-apoptotic function of PDCD4 was validated in hepatocyte cell lines, as PDCD4 knockout inhibited while PDCD4 overexpression promoted APAP-induced apoptosis (Fig. 4j, k and Supplementary Fig. S4,E). As PDCD4 is known to repress internal ribosome entry site-mediated translation of BCL-X_L_ and suppress its expression^25^, the decreased BCL-X_L_ expression was also determined above in the miR-199a-3p knockout hepatocyte cell lines and liver tissues (Supplementary Fig. S3E–H). Altogether, these data suggest that *miR-199a-2* knockout may increase PDCD4 to inhibit BCL-X_L_ expression, so as to enhance the activation of hepatic mitochondrial apoptosis pathway.

### miR-199a-3p suppresses hepatocyte apoptosis by targeting PDCD4

We next examined whether miR-199a-3p-suppressed hepatocyte apoptosis was dependent on PDCD4. miR-199a-3p mimics was transfected into the control and PDCD4 knockout hepatocyte cell lines, and miR-199a-3p-mediated inhibition of APAP-induced cleaved caspase-3 was abolished in PDCD4 knockout cell lines (Supplementary Fig. S5A). Moreover, PDCD4 was also overexpressed in control and *miR*-*199a*-*2* knockout hepatocyte cell lines, and miR-199a-3p knockout-mediated increase of cleaved caspase-3 was abolished in PDCD4 overexpressed cell lines (Supplementary Fig. S5B). Thus, the miR-199a-3p-mediated inhibition of hepatocyte apoptosis was dependent on PDCD4 in vitro.

**Fig. 5:**
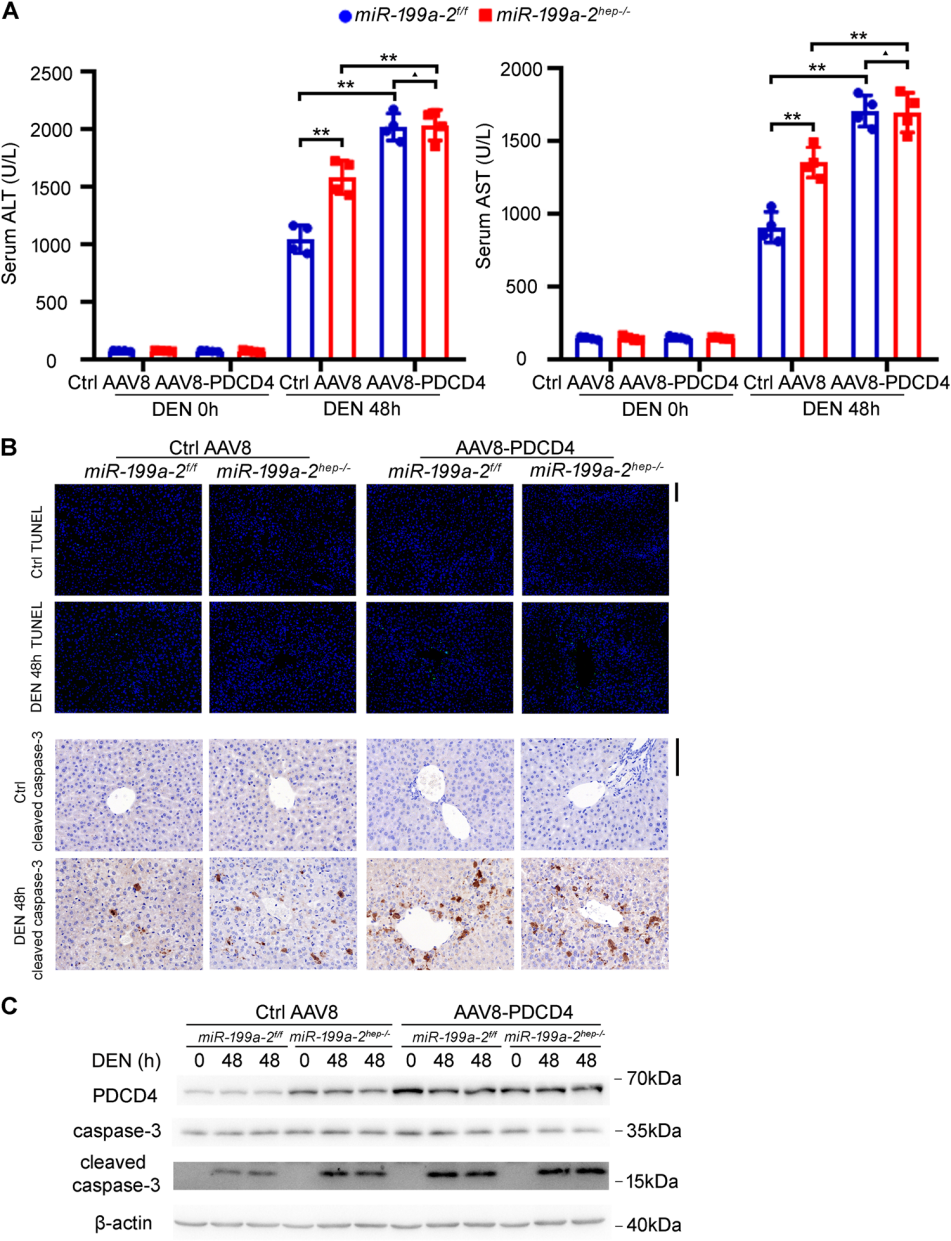
miR-199a-3p inhibits DEN-induced hepatocyte apoptosis and hepatic injury by targeting PDCD4 in vivo. Control AAV8 or AAV8-PDCD4 were injected into *miR*-*199a*-*2*^*f/f*^ or *miR-199a*-*2*^*hep-/-*^ mice through tail vein at a dose of 1 × 10^12^ vg per mouse, and DEN (100 mg/kg) was injected 4 weeks later. Serum ALT and AST (*n* = 4) (**a**), TUNEL and cleaved caspase-3 IHC staining (**b**), and cleaved caspase-3 blot (**c**) were measured in the indicated time points post DEN administration. Data are shown as mean ± SD or typical photographs of one representative experiment. Similar results were obtained in three independent experiments. ^▲^*P* > 0.05; **P < 0.01.

**Fig. 6:**
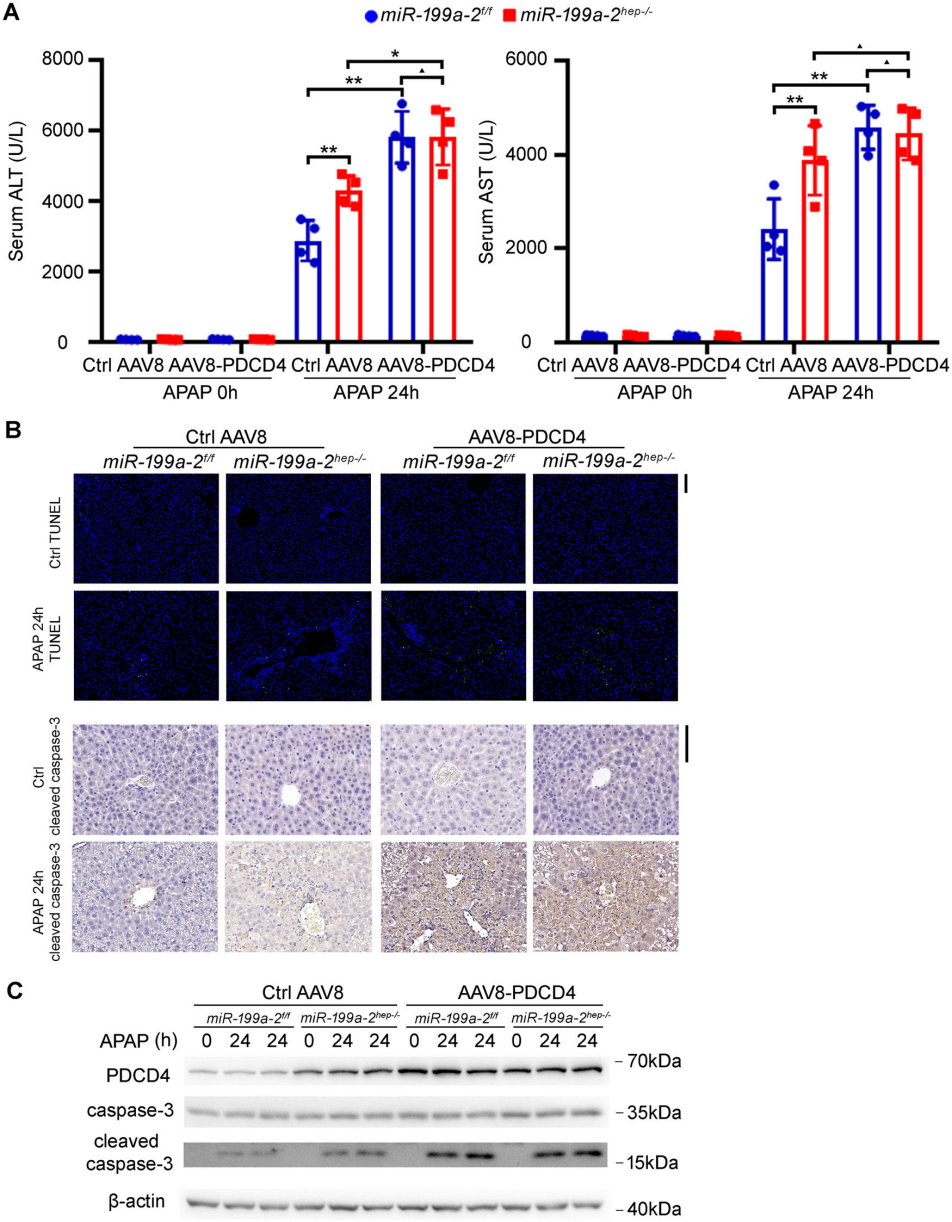
miR-199a-3p inhibits APAP-induced hepatocyte apoptosis and hepatic injury by targeting PDCD4 in vivo. Control AAV8 or AAV8-PDCD4 were injected into *miR*-*199a*-*2*^*f/f*^ or *miR-199a*-*2*^*hep-/-*^ mice through tail vein at a dose of 1 × 10^12^ vg per mouse, and APAP (400 mg/kg) was injected 4 weeks later. Serum ALT and AST (*n* = 4) (**a**), TUNEL and cleaved caspase-3 IHC staining (**b**), and cleaved caspase-3 blot (**c**) were measured in the indicated time points post APAP administration. Data are shown as mean ± SD or typical photographs of one representative experiment. Similar results were obtained in three independent experiments. ^▲^*P* > 0.05; *P < 0.05; **P < 0.01.

To analyze whether miR-199a-3p-suppressed hepatocyte apoptosis was dependent on PDCD4 in vivo, PDCD4 was overexpressed in the liver of *miR*-*199a*-*2*^*f/f*^ and *miR*-*199a*-*2*^*hep-/-*^ mice through AAV8-mediated gene delivery. miR-199a-3p knockout-mediated increase of DEN-induced hepatocyte apoptosis and hepatic injury, suggested by the elevated serum ALT and AST, TUNEL and cleaved caspase-3 staining, and cleaved caspase-3 blot, were abolished in the PDCD4 overexpressed livers (Fig. 5a–c). Similarly, in the APAP-induced hepatic injury model, miR-199a-3p knockout-promoted hepatocyte apoptosis and hepatic injury were also abolished in the PDCD4 overexpressed livers (Fig. 6a–c). Altogether, these data determine that miR-199a-3p suppresses hepatocyte apoptosis and hepatic injury by targeting PDCD4.

## Discussion

Hepatocyte death and liver injury cause the release of DAMPs and the induced liver inflammation, which subsequently stimulate compensatory hepatocyte proliferation and repair of liver function. However, the repeated vicious cycles of chronic injury, inflammation, and hepatocyte proliferation cause liver cirrhosis and hepatocarcinogenesis^27^. During this process, hepatocyte death and liver injury play critical roles in this cycle, and hepatocyte apoptosis-initiated inflammatory circumstances are determined to induce the development of HCC^28^. Thus, understanding the intracellular mechanisms responsible for the control of hepatocyte apoptosis is important in future investigation of the strategies for the intervention of HCC. Based on our previous study that miR-199a-3p was abundantly expressed in the liver, we found here that hepatic miR-199a-3p was able to inhibit the hepatocyte apoptosis and liver injury, thus suppressing the subsequent hepatocarcinogenesis. Moreover, the apoptosis inhibition mediated by hepatic miR-199a-3p may also suggest its role in maintaining liver function during acute liver injury. Although hepatic *miR-199a-2* knockout does not lead to spontaneous liver injury, it promotes hepatocyte apoptosis upon DEN injection, while other aspects such as hepatocyte DNA damage and compensatory proliferation were not influenced. Together with the pivotal role of hepatocyte apoptosis in driving HCC carcinogenesis, hepatic *miR-199a-2* knockout-promoted hepatocarcinogenesis is at least in part mediated by the increased hepatocyte apoptosis upon DEN injection.

The development of HCC could be divided into two main courses, which is the first carcinogenesis and then progression. Currently, a set of miRNAs have been determined to play important roles in HCC progression, including enhanced proliferation, apoptosis inhibition, cell cycle progression, and promoted migration and invasion^14–16^. On the other point, the functions and underlying mechanisms of miRNAs in hepatocarcinogenesis are still poorly understood, especially determined in vivo. Using miR-122 or miR-148a knockout mice, these two miRNAs have been implicated in hepatic lipid metabolism and the related hepatocarcinogenesis^17,29^. However, the roles of miRNAs in regulating hepatocyte apoptosis and the related hepatocarcinogenesis remain unknown. Here, hepatic abundant miR-199a-3p was determined to protect hepatocytes from apoptosis during liver injury, thus suppressing the related hepatocarcinogenesis, suggesting the important regulatory roles of miRNAs in modulating hepatocyte death and liver injury during hepatocarcinogenesis. Moreover, the potential regulatory roles of miRNAs in the induced hepatic inflammation and compensatory hepatocyte proliferation following liver injury during hepatocarcinogenesis are also important issues, which needs to be further investigated.

The inhibitory role of miR-199a-3p in hepatocarcinogenesis was determined using DEN and DEN plus CCl_4_-induced mouse hepatocarcinogenesis models. In addition, genetic models such as *Tak1*^*hep-/-*^ or *Mdr2*^*-/-*^ mice representing chronic liver injury, MUP-uPA transgenic mice representing continuous hepatic ER stress, and Stelic Animal Model (STAM) representing metabolic disorders-induced HCC, were also commonly used in the study of molecular regulatory mechanisms in hepatocarcinogenesis^30–33^. It was suggested that the HCC tissues form STAM model, induced by streptozocin and high-fat diet (HFD), were the most similar to human HCC by transcriptome analysis^34^. Thus, the potential roles of hepatic abundant miR-199a-3p in the STAM metabolic hepatocarcinogenesis model, including its roles in liver metabolic diseases such as NAFLD and NASH, are still unknown up to now, which will be investigated in the future.

We previously reported that miR-199a-3p was abundantly expressed in normal liver but markedly decreased in HCC tissues, and decreased miR-199a-3p promoted HCC survival and growth. In this work, we found that hepatic miR-199a-3p knockout promoted hepatocarcinogenesis, and miR-199a-3p knockout enhanced hepatocyte apoptosis. Together with these data, two important scientific questions are raised. First, why does decreased miR-199a-3p enhance apoptosis in hepatocytes while promote survival in HCC? An easy explanation may be that miR-199a-3p functions differently in different cell types by targeting different genes expression. PAK4, the target of miR-199a-3p we found in HCC, was not detected by the proteomic analysis in miR-199a-3p deficient hepatocytes in this work. However, the underlying mechanisms responsible for the different main targets of a single miRNA in different cell types still need intensive investigation, and these mechanisms may unveil the different modes of interaction among molecules between benign hepatocyte and malignant HCC. Second, as miR-199a-3p expression is markedly decreased in HCC, whether miR-199a-3p is decreased in hepatocytes first and then promote hepatocyte apoptosis and liver injury, or it is decreased only in established HCC to promote HCC survival and progression? The answer to this question may need the identification of detailed transformation process from hepatocyte to HCC, and examination of miR-199a-3p expression and change of function in the interim cell types between hepatocytes and HCC. Additionally, the possibility also exists that miR-199a-3p is abundantly expressed in hepatocytes and maintain their survival and liver function before HCC formation, while only decreased in established HCC, when its function changes and decreased miR-199a-3p promotes HCC survival and progression. These two questions may raise interesting future works in this issue.

Another interesting point is that there are three gene loci to express mature miR-199-3p, including *miR-199a-1*, *miR-199a-2*, and *miR-199b*, in the genome of both human and mouse as miRNAs are evolutionarily conserved non-coding RNAs. In the liver, we determined that miR-199-3p was mainly from *miR-199a-2*, because miR-199-3p is not detectable when *miR-199a-2* is knocked out. In some other organs such as spleen and kidney, miR-199-3p is also from *miR-199a-2* gene, which is identified in *miR-199a-2* knockout mice (data not shown), and we presume that miR-199a-3p may work similarly to suppress apoptosis and maintain function in these organs. However, deletion of *miR-199a-2* does not reduce miR-199-3p expression in lung and colon, while *miR-199a-1* knockout does (Data not show), suggesting that miR-199 is expressed by different gene locus in different organs. As miR-199 was reported to be dysregulated in cancer from all these organs^35–37^, the corresponding mechanisms responsible for the dysregulation of miR-199 may be also different. Moreover, in mouse islets, miR-199a-3p is from both *miR-199a-1* and *miR-199a-2*, but not *miR-199b*, and the pri-miR-199a-1 is about 30% more than pri-miR-199a-2, whose expression are regulated by glucose metabolism and calcium influx^38^. Thus, why miR-199-3p is expressed from different gene locus in different organs and the underlying biological significance are interesting topics, which still need further exploration.

For the roles of PDCD4 in carcinogenesis, previous reports suggested that PDCD4 knockout mice could spontaneously develop lymphoma around 85 weeks, which may be mediated by the upregulated IL-4 and IL-10 produced by *pdcd4*^-/-^ immune cells^39^. Besides, PDCD4 knockout also promoted AOM plus DSS-induced colorectal cancer by the mechanism that PDCD4 deficiency increased the macrophage-secreted IL-6 induced by DSS and elevated the epithelial cell susceptibility to IL-6/STAT3 pathway-mediated cell proliferation during malignant transformation^40^. In this work, we observed the increased level of PDCD4 in *miR-199a-2*^*hep-/-*^ mice, which enhanced hepatocyte apoptosis induced by DEN and APAP, and the promoted hepatocarcinogenesis in *miR-199a-2*^*hep-/-*^ mice induced by DEN and DEN plus CCl_4_. In different types of cells, the functions of PDCD4 are probably to be different, which may be mediated by the different PDCD4-regulated gene expression and signaling activation. This description may explain the probable different roles of PDCD4 in lymphocytes, macrophages, epithelium, and hepatocytes. Although we determined here that miR-199a-3p targets PDCD4 to inhibit hepatocyte apoptosis and then suppress hepatocarcinogenesis, the direct evidence of PDCD4 in hepatocarcinogenesis is still not determined in this study, and we will keep focusing on this issue in our future study.

## Materials and methods

### Mice and cell lines

*miR*-*199a*-*1* conventional knockout mice were constructed by Model Animal Research Center of Nanjing University (Nanjing, China). *miR*-*199a*-*2* conventional knockout mice were constructed by Beijing VIEWSOLID BIOTECH Ltd (Beijing, China). *miR*-*199a*-*2*^*f/f*^ mice were constructed by Shanghai Model Organism Center (Shanghai, China). Identification of mouse genotype was carried out by PCR analysis using genomic DNA purified from tail tissues (Supplementary Table S1)^41^. All animal experiments were undertaken in accordance with National Institutes of Health’s *Guide for the Care and Use of Laboratory Animals* with the approval of the institutional research ethics committee of Second Military Medical University, Shanghai, China. HL-7702 and BNL CL.2 cell lines were obtained from cell bank of Chinese Academy of Sciences (Shanghai, China). All cell lines have been authenticated using STR profiling and tested for mycoplasma contamination by Genechem (Shanghai, China). The knockout cell lines were constructed using CRISPR/Cas9 method (Supplementary Table S2)^42^. HL-7702 was cultured in RPMI 1640 with 10% Fetal Bovine Serum (FBS), and BNL CL.2 was cultured in dulbecco’s modified eagle medium (DMEM) with 10% FBS and 1% non-essential animo acid (NAA).

### Reagents

Antibodies specific to caspase-3 (9662), cleaved caspase-3 (9661), caspase-8 (4790), human cleaved caspase-8 (9496), mouse cleaved caspase-8 (9429), BAK (12105), BAX (2772), BCL-2 (3498), BCL-X_L_ (2764), PDCD4 (9535), and horseradish peroxidase-coupled secondary antibodies (7074 and 7076) were from Cell Signaling Technology (Danvers, MA). Antibody specific to β-actin (A5441), DEN (N0258), DHE (D7008), CHX (239764), LPS (L3024) and MEM NAA (M7145) were from Sigma-Aldrich (St. Louis, MO). Antibody specific to cytochrome C (sc-13156) was from Santa Cruz Biotechnology (CA, USA). Recombinant human TNF-α (300-01A) and murine TNF-α (315-01 A) were from Pepro Tech (Rocky Hill, NJ). TUNEL assay kit (11684817910) was from Roche (Shanghai, China). CCl_4_ (CAS: 56-23-5, C805329) was from MAKLIN (Shanghai, China). Olive oil (CAS: 8001-25-0, A502795-0100), APAP (CAS: 103-90-2, A506808), and D-hanks buffer (B548148-0500) were from Sangon Biotech (Shanghai, China). Cell mitochondria isolation kit (C3601) and tissue mitochondria isolation kit (C3606) were from Beyotime (Shanghai, China). Percoll solution (17-0891-01) was from GE Healthcare Life Science (Little Chalfont, UK). Type IV collagenase (LS004140) was from Worthington Biochemical Corporation (Lakewood, NJ). Protease inhibitor cocktail (539134-1SML) and apoptosis assay kit (PF032) were from Calbiochem (Darmstadt, Germany).

### Establishment of hepatocarcinogenesis model

To construct DEN-induced hepatocarcinogenesis model, the 2-week-old male mice were injected with 25 mg/kg body weight DEN intraperitoneally^43^. Mice were sacrificed 8 months later and tumors were analyzed. As for DEN plus CCl_4_ model, the 2-week-old male mice were injected with 25 mg/kg body weight DEN intraperitoneally, and 4 weeks later, mice were injected with CCl_4_ (0.5 ml/kg body weight, dissolved in olive oil at a ratio of 1:3) weekly lasting for 15 weeks^19^. Mice were sacrificed 8 weeks after last CCl_4_ injection.

### Establishment of acute hepatic injury model

To constructed DEN-induced acute hepatic injury model, 8-week-old male mice were injected with DEN (100 mg/kg) intraperitoneally and mice were sacrificed according to the time points. As for APAP-induced acute hepatic injury model, eight-week-old male mice were fasted overnight (16 h) and then injected with APAP (400 mg/kg) intraperitoneally. Mice were fed immediately after injection and sacrificed as indicated. Serum ALT and AST were measured by automatic biochemical analyzer FDC-7000i (Shanghai, China).

### In vivo AAV8 administration

The rAAV (serotype 8) vector expressing PDCD4 under promoter CAG was constructed as previously described^18^. For AAV8 administration, 1 × 10^12^ vg AAV8 per mouse in 200 μl saline buffer was injected into the mice through tail vein. Four weeks later, the protein expression of PDCD4 was measured by Western blot.

### Isolation of primary hepatocytes

Primary hepatocytes were isolated from mouse liver using two-step collagenase perfusion procedure^44^. Mice were anesthetized and first perfused with 45 ml D-hanks buffer and then with type IV collagenase digestion (1 mg/ml) for 5 min at a rate of 2 ml/min through the portal vein. After digestion, the liver was excised, minced, filtered through 70-micron membrane, spin for 5 min at 50 × *g*, and purified using 50% Percoll solution. The obtained hepatocytes were resuspended in DMEM, and trypan blue exclusion assays indicated that cell viability was >95%. Primary hepatocytes were cultured in DMEM with 10% FBS and 1% Penicillin-Streptomycin.

### RNA isolation and quantitative PCR analysis

Total RNA was extracted from mouse liver tissues or cell lines using RNAiso Plus reagent (Takara, Dalian, China) according the recommended protocol. Real-time quantitative RT-PCR (qRT-PCR) analysis was performed using LightCycler 2.0 (Roche, Switzerland) and SYBR RT-PCR kit (Takara) as previously described^18^. The relative expression level of the individual genes was normalized to that of internal control by using 2^-ΔΔCt^ cycle threshold method in each sample (Supplementary Table S3)^45^.

### Western blot

Cells or tissues were lysed on ice with cell lysis buffer (Cell Signaling Technology) supplemented with protease inhibitor cocktail (Calbiochem) at a ratio of 1:200. Protein concentrations of the extracts were measured with bicinchoninic acid (BCA) assay (Pierce). Equal amount of the extracts was loaded to sodium dodecyl sulfate polyacrylamide gel electrophoresis and transferred onto nitrocellulose membrane for immunoblot analysis as described previously^20^.

### Histology

For HE, IHC and TUNEL, liver tissues were fixed in 4% paraformaldehyde for 48 h, embedded by paraffin and sliced up into 5 μm thick sections, which were processed to HE, IHC, and TUNEL as kit protocols described. For reactive oxygen species (ROS) measurement, fresh liver tissues were embedded by Tissue-Tek^®^ O.C.T compound and immediately sliced up into liver sections. The slices were incubated in DHE (1 μM) for 30 min. After washing with PBS for three times, the red fluorescence was measured by fluorescence microscope.

### Dual-luciferase reporter assay

The PDCD4 luciferase reporter was made by amplifying the mouse *pdcd4* mRNA by PCR and cloned into the 3ʹUTR region of pMIR-promoter-Firefly plasmids. The luciferase reporter plasmids, RL-TK-Renilla plasmids, and indicated miRNAs (final concentration 20 nM) were co-transfected into HEK 293T cells. After 24 h, the activities of Firefly and Renilla luciferases were measured using the Dual-Luciferase Reporter Assay System (Promega) as previously described^46^. Data was normalized for transfection efficiency by dividing Firefly luciferase activity with that of Renilla luciferase.

### Flow cytometry

The control and knockout cells were treated with APAP (10 mM) for 24 h. Cells were collected and labeled by apoptosis assay kit and subjected to flow cytometry analysis on LSRII. Data were analyzed using FACSDiva software (Becton Dickinson).

### Statistical analysis

As indicated in the figure legends, sample size of the experiments depended on the assay type. There were no blind experiments for the investigators both in cells and mice experiments. The mice were randomly distributed to the experimental group. For all groups that are statistically compared, the variance within each group was similar. Data are shown as mean ± SD from one representative of three independent experiments. Statistical comparisons between experimental groups were analyzed by unpaired Student’s *t*-test or chi-square test in GraphPad Prism 8.0, and a two-tailed *P* < 0.05 was taken to indicate statistical significance.

## Supplementary information

Author Confirmation File

Supplementary Information

Supplementary tables

Figure S1

Figure S2

Figure S3

Figure S4

Figure S5
