# Supplementary material for: microRNA-199a-3p inhibits hepatic apoptosis and hepatocarcinogenesis by targeting PDCD4
Source: Oncogenesis. 2020 Oct 24;9(10):95. doi: 10.1038/s41389-020-00282-y (PMC7585580; doi:10.1038/s41389-020-00282-y)
Supplement: Supplementary file 1 — Author Confirmation File [file 41389_2020_282_MOESM1_ESM.pdf]

Author Confirmation File for

microRNA-199a-3p inhibits hepatic apoptosis and hepatocarcinogenesis by targeting PDCD4

Zhenyang Li, Ye Zhou, Liyuan Zhang, Kaiwei Jia, Suyuan Wang, Mu Wang, Nan Li, Yizhi Yu, Xuetao Cao, and Jin Hou

National Key Laboratory of Medical Immunology & Institute of Immunology, Second Military Medical University, Shanghai 200433, China

These authors contributed equally: Zhenyang Li, Ye Zhou, Liyuan Zhang

Correspondence: Jin Hou (houjin@immunol.org) or Xuetao Cao (caoxt@immunol.org)

National Key Laboratory of Medical Immunology & Institute of Immunology, Second Military Medical University, 800 Xiangyin Road, Shanghai 200433, China. Phone: (+86-21) 5562 0605; Fax: (+86-21) 6538 2502

Author Emails:

Zhenyang Li (lzy910722@126.com), Ye Zhou (yzhou1989@126.com), Liyuan Zhang (zhangliyuanapril@163.com), Kaiwei Jia (15721570382@163.com), Suyuan Wang (15021581996@163.com), Mu Wang (wangmukl@gmail.com), Nan Li (linan@immunol.org), Yizhi Yu (yuyz@immunol.org), Xuetao Cao (caoxt@immunol.org), and Jin Hou (houjin@immunol.org)

The Email of Authorship sent to each author:

Dear all co-authors:

For our manuscript entitled “microRNA-199a-3p inhibits hepatic apoptosis and hepatocarcinogenesis by targeting PDCD4”, which is submitted to Oncogenesis (ONCSIS-20-0261RR), this manuscript has been accepted by Oncogenesis currently. This email was to confirm the authorship of this manuscript.

The listed authors are: “Zhenyang Li, Ye Zhou, Liyuan Zhang, Kaiwei Jia, Suyuan Wang, Mu Wang, Nan Li, Yizhi Yu, Xuetao Cao, and Jin Hou”. These authors are all from “National Key Laboratory of Medical Immunology & Institute of Immunology, Second Military Medical University, Shanghai 200433, China”. The authors “Zhenyang Li, Ye Zhou, Liyuan Zhang” are co-first authors. The authors “Xuetao Cao (caoxt@immunol.org), and Jin Hou (houjin@immunol.org) are co-correspondence.

Please confirm the authorship listed above, and reply this email. Thank you very much for your cooperation.

Yours sincerely,

Jin Hou, Ph.D.

Professor, Institute of Immunology

National Key Laboratory of Medical Immunology

Second Military Medical University

800 Xiangyin Road, Shanghai 200433, China

Phone: (+86 21) 5562 0605; Fax: (+86 21) 6538 2502

Email: houjin@immunol.org

邮箱助手 houjin

houjin 查看相关会话 18:41

收件人: houjin caoxt@immunol.org lzy910722 yzhou1989 zhangliyuanapril 15721570382 15021581996 wangmukl linan@immunol.org yuyz

✓ 发送成功，查看读信状态 撤回邮件

Please confirm the authorship of our manuscript "microRNA-199a-3p inhibits hepatic apoptosis and hepatocarcinogenesis by targeting PDCD4"

Dear all co-authors:

For our manuscript entitled "microRNA-199a-3p inhibits hepatic apoptosis and hepatocarcinogenesis by targeting PDCD4", which is submitted to *Oncogenesis* (ONCSIS-20-0261RR), this manuscript has been accepted by *Oncogenesis* currently. This email was to confirm the authorship of this manuscript.

The listed authors are: "Zhenyang Li, Ye Zhou, Liyuan Zhang, Kaiwei Jia, Suyuan Wang, Mu Wang, Nan Li, Yizhi Yu, Xuetao Cao, and Jin Hou". These authors are all from "National Key Laboratory of Medical Immunology & Institute of Immunology, Second Military Medical University, Shanghai 200433, China". The authors "Zhenyang Li, Ye Zhou, Liyuan Zhang" are co-first authors. The authors "Xuetao Cao (caoxt@immunol.org), and Jin Hou (houjin@immunol.org) are co-correspondence.

Please confirm the authorship listed above, and reply this email. Thank you very much for your cooperation.

Yours sincerely,

Jin Hou, Ph.D.
Professor, Institute of Immunology
National Key Laboratory of Medical Immunology
Second Military Medical University
800 Xiangyin Road, Shanghai 200433, China
Phone: (+86 21) 5562 0605; Fax: (+86 21) 6538 2502
Email: houjin@immunol.org

1, Confirmation from Zhenyang Li

邮箱助手 houjin

李振洋 查看相关会话 19:45

收件人: houjin

Re: Please confirm the authorship of our manuscript "microRNA-199a-3p inhibits hepatic apoptosis and hepatocarcinogenesis by targeting PDCD4"

正文中含有来自其他站点的图片或链接，如遇提示输入帐户名和密码，切勿提交！ 了解钓鱼风险

Okay, I have confirmed that the authorship list is without problems.

Zhenyang Li

Institute of Immunology

National Key Laboratory of Medical Immunology

Second Military Medical University

800 Xiangyin Road, Shanghai 200433, China

Phone: (+86 21) 5562 0605; Fax: (+86 21) 6538 2502

Email: lzy910722@126.com

李振洋

lzy910722@126.com

签名由 网易邮箱大师 定制

On 9/28/2020 18:41，houjin<houjin@immunol.org> wrote:

——— 隐藏引用的内容 ———

Dear all co-authors:

For our manuscript entitled "microRNA-199a-3p inhibits hepatic apoptosis and hepatocarcinogenesis by targeting PDCD4", which is submitted to *Oncogenesis* (ONCSIS-20-0261RR), this manuscript has been accepted by *Oncogenesis* currently. This email was to confirm the authorship of this manuscript.

The listed authors are: "Zhenyang Li, Ye Zhou, Liyuan Zhang, Kaiwei Jia, Suyuan Wang, Mu Wang, Nan Li, Yizhi Yu, Xuetao Cao, and Jin Hou". These authors are all from "National Key Laboratory of Medical Immunology & Institute of Immunology, Second Military Medical University, Shanghai 200433, China". The authors "Zhenyang Li, Ye Zhou, Liyuan Zhang" are co-first authors. The authors "Xuetao Cao (caoxt@immunol.org), and Jin Hou (houjin@immunol.org) are co-correspondence.

Please confirm the authorship listed above, and reply this email. Thank you very much for your cooperation.

Yours sincerely,

Jin Hou, Ph.D.
Professor, Institute of Immunology
National Key Laboratory of Medical Immunology
Second Military Medical University
800 Xiangyin Road, Shanghai 200433, China
Phone: (+86 21) 5562 0605; Fax: (+86 21) 6538 2502
Email: houjin@immunol.org

2, Confirmation from Ye Zhou

邮箱助手 houjin

周烨 查看相关会话 19:06

收件人: houjin

Re: Please confirm the authorship of our manuscript "microRNA-199a-3p inhibits hepatic apoptosis and hepatocarcinogenesis by targeting PDCD4"

正文中含有来自其他站点的图片或链接，如遇提示输入帐户名和密码，切勿提交！了解钓鱼风险

Yes, the authorship list is right.

Ye Zhou
National Key Laboratory of Medical Immunology
Second Military Medical University
800 Xiangyin Road, Shanghai 200433, China

周烨
邮箱：yzhou1989@126.com

签名由 网易邮箱大师 定制

On 09/28/2020 18:41, houjin wrote:

——— 隐藏引用的内容 ———

Dear all co-authors:

For our manuscript entitled "microRNA-199a-3p inhibits hepatic apoptosis and hepatocarcinogenesis by targeting PDCD4", which is submitted to *Oncogenesis* (ONCSIS-20-0261RR), this manuscript has been accepted by *Oncogenesis* currently. This email was to confirm the authorship of this manuscript.

The listed authors are: "Zhenyang Li, Ye Zhou, Liyuan Zhang, Kaiwei Jia, Suyuan Wang, Mu Wang, Nan Li, Yizhi Yu, Xuetao Cao, and Jin Hou". These authors are all from "National Key Laboratory of Medical Immunology & Institute of Immunology, Second Military Medical University, Shanghai 200433, China". The authors "Zhenyang Li, Ye Zhou, Liyuan Zhang" are co-first authors. The authors "Xuetao Cao (caoxt@immunol.org), and Jin Hou (houjin@immunol.org) are co-correspondence.

Please confirm the authorship listed above, and reply this email. Thank you very much for your cooperation.

Yours sincerely,

Jin Hou, Ph.D.
Professor, Institute of Immunology
National Key Laboratory of Medical Immunology
Second Military Medical University
800 Xiangyin Road, Shanghai 200433, China
Phone: (+86 21) 5562 0605; Fax: (+86 21) 6538 2502
Email: houjin@immunol.org

3, Confirmation from Liyuan Zhang

邮箱助手 houjin

张力元 查看相关会话 19:08

收件人: houjin

Re: Please confirm the authorship of our manuscript "microRNA-199a-3p inhibits hepatic apoptosis and hepatocarcinogenesis by targeting PDCD4"

正文中含有来自其他站点的图片或链接，如遇提示输入帐户名和密码，切勿提交！了解钓鱼风险

I confirm that the authorship is okay.

zhangliyuanapril
邮箱：zhangliyuanapril@163.com

Signature is customized by Netease Mail Master

On 09/28/2020 18:41, houjin wrote:

——— 隐藏引用的内容 ———

Dear all co-authors:

For our manuscript entitled “microRNA-199a-3p inhibits hepatic apoptosis and hepatocarcinogenesis by targeting PDCD4”, which is submitted to *Oncogenesis* (ONCSIS-20-0261RR), this manuscript has been accepted by *Oncogenesis* currently. This email was to confirm the authorship of this manuscript.

The listed authors are: “Zhenyang Li, Ye Zhou, Liyuan Zhang, Kaiwei Jia, Suyuan Wang, Mu Wang, Nan Li, Yizhi Yu, Xuetao Cao, and Jin Hou”. These authors are all from “National Key Laboratory of Medical Immunology & Institute of Immunology, Second Military Medical University, Shanghai 200433, China”. The authors “Zhenyang Li, Ye Zhou, Liyuan Zhang” are co-first authors. The authors “Xuetao Cao (caoxt@immunol.org), and Jin Hou (houjin@immunol.org) are co-correspondence.

Please confirm the authorship listed above, and reply this email. Thank you very much for your cooperation.

Yours sincerely,

Jin Hou, Ph.D.
Professor, Institute of Immunology
National Key Laboratory of Medical Immunology
Second Military Medical University
800 Xiangyin Road, Shanghai 200433, China
Phone: (+86 21) 5562 0605; Fax: (+86 21) 6538 2502
Email: houjin@immunol.org

4, Confirmation from Kaiwei Jia

邮箱助手 houjin

郑凯威 查看相关会话 20:03

收件人: houjin

Re:Please confirm the authorship of our manuscript "microRNA-199a-3p inhibits hepatic apoptosis and hepatocarcinogenesis by targeting PDCD4"

OK, I have confirmed the authorship which is right.

Kaiwei Jia
Institute of Immunology
National Key Laboratory of Medical Immunology
Second Military Medical University
800 Xiangyin Road, Shanghai 200433, China
Phone: (+86 21) 5562 0605; Fax: (+86 21) 6538 2502
Email: 15721570382@163.com

At 2020-09-28 18:41:30, "houjin" <houjin@immunol.org> wrote:

——— 隐藏引用的内容 ———

Dear all co-authors:

For our manuscript entitled "microRNA-199a-3p inhibits hepatic apoptosis and hepatocarcinogenesis by targeting PDCD4", which is submitted to *Oncogenesis* (ONCSIS-20-0261RR), this manuscript has been accepted by *Oncogenesis* currently. This email was to confirm the authorship of this manuscript.

The listed authors are: "Zhenyang Li, Ye Zhou, Liyuan Zhang, Kaiwei Jia, Suyuan Wang, Mu Wang, Nan Li, Yizhi Yu, Xuetao Cao, and Jin Hou". These authors are all from "National Key Laboratory of Medical Immunology & Institute of Immunology, Second Military Medical University, Shanghai 200433, China". The authors "Zhenyang Li, Ye Zhou, Liyuan Zhang" are co-first authors. The authors "Xuetao Cao (caoxt@immunol.org), and Jin Hou (houjin@immunol.org) are co-correspondence.

Please confirm the authorship listed above, and reply this email. Thank you very much for your cooperation.

Yours sincerely,

Jin Hou, Ph.D.
Professor, Institute of Immunology
National Key Laboratory of Medical Immunology
Second Military Medical University
800 Xiangyin Road, Shanghai 200433, China
Phone: (+86 21) 5562 0605; Fax: (+86 21) 6538 2502
Email: houjin@immunol.org

5, Confirmation from Suyuan Wang

邮箱助手 houjin

15021581996 查看相关会话 19:08

收件人: houjin 详情

抄 送: houjin caoxt@immunol.org lzy910722 yzhou1989 zhangliyuanapril 15721570382 wangmukl linan@immunol.org yuyz

Re: Please confirm the authorship of our manuscript "microRNA-199a-3p inhibits hepatic apoptosis and hepatocarcinogenesis by targeting PDCD4"

正文中含有来自其他站点的图片或链接，如遇提示输入帐户名和密码，切勿提交！了解钓鱼风险

The authorship list is okay.

王素媛

邮箱：15021581996@163.com

签名由 网易邮箱大师 定制

On 09/28/2020 18:41, houjin wrote:

——— 隐藏引用的内容 ———

Dear all co-authors:

For our manuscript entitled "microRNA-199a-3p inhibits hepatic apoptosis and hepatocarcinogenesis by targeting PDCD4", which is submitted to *Oncogenesis* (ONCSIS-20-0261RR), this manuscript has been accepted by *Oncogenesis* currently. This email was to confirm the authorship of this manuscript.

The listed authors are: "Zhenyang Li, Ye Zhou, Liyuan Zhang, Kaiwei Jia, Suyuan Wang, Mu Wang, Nan Li, Yizhi Yu, Xuetao Cao, and Jin Hou". These authors are all from "National Key Laboratory of Medical Immunology & Institute of Immunology, Second Military Medical University, Shanghai 200433, China". The authors "Zhenyang Li, Ye Zhou, Liyuan Zhang" are co-first authors. The authors "Xuetao Cao (caoxt@immunol.org), and Jin Hou (houjin@immunol.org) are co-correspondence.

Please confirm the authorship listed above, and reply this email. Thank you very much for your cooperation.

Yours sincerely,

Jin Hou, Ph.D.
Professor, Institute of Immunology
National Key Laboratory of Medical Immunology
Second Military Medical University
800 Xiangyin Road, Shanghai 200433, China
Phone: (+86 21) 5562 0605; Fax: (+86 21) 6538 2502
Email: houjin@immunol.org

6, Confirmation from Mu Wang

邮箱助手 houjin

王沐

收件人: houjin

查看相关会话 19:43

Re: Please confirm the authorship of our manuscript "microRNA-199a-3p inhibits hepatic apoptosis and hepatocarcinogenesis by targeting PDCD4"

The authorship is confirmed and it is right.

Mu Wang
Institute of Immunology
National Key Laboratory of Medical Immunology
Second Military Medical University
800 Xiangyin Road, Shanghai 200433, China
Phone: (+86 21) 5562 0605; Fax: (+86 21) 6538 2502
Email: wangmukl@gmail.com

houjin <houjin@immunol.org> 于2020年9月28日周一 下午6:41写道:

——— 隐藏引用的内容 ———

Dear all co-authors:

For our manuscript entitled "microRNA-199a-3p inhibits hepatic apoptosis and hepatocarcinogenesis by targeting PDCD4", which is submitted to *Oncogenesis* (ONCSIS-20-0261RR), this manuscript has been accepted by *Oncogenesis* currently. This email was to confirm the authorship of this manuscript.

The listed authors are: "Zhenyang Li, Ye Zhou, Liyuan Zhang, Kaiwei Jia, Suyuan Wang, Mu Wang, Nan Li, Yizhi Yu, Xuetao Cao, and Jin Hou". These authors are all from "National Key Laboratory of Medical Immunology & Institute of Immunology, Second Military Medical University, Shanghai 200433, China". The authors "Zhenyang Li, Ye Zhou, Liyuan Zhang" are co-first authors. The authors "Xuetao Cao (caoxt@immunol.org), and Jin Hou (houjin@immunol.org) are co-correspondence.

Please confirm the authorship listed above, and reply this email. Thank you very much for your cooperation.

Yours sincerely,

Jin Hou, Ph.D.
Professor, Institute of Immunology
National Key Laboratory of Medical Immunology
Second Military Medical University
800 Xiangyin Road, Shanghai 200433, China
Phone: (+86 21) 5562 0605; Fax: (+86 21) 6538 2502
Email: houjin@immunol.org

7, Confirmation from Nan Li

邮箱助手 houjin

linan@immunol.org 查看相关会话 19:32

收件人: houjin

回复：Please confirm the authorship of our manuscript "microRNA-199a-3p inhibits hepatic apoptosis and hepatocarcinogenesis by targeting PDCD4"

The authorship listed is confirmed.

Nan Li

——— 隐藏引用的内容 ———

发件人：houjin <houjin@immunol.org>
发送时间：2020年9月28日(星期一) 18:41
收件人：houjin <houjin@immunol.org>; caoxt@immunol.org <caoxt@immunol.org>; lzy910722 <lzy910722@126.com>; 周烨 <yzhou1989@126.com>; zhangliyuanapril <zhangliyuanapril@163.com>; 15721570382 <15721570382@163.com>; 15021581996 <15021581996@163.com>; wangmukl <wangmukl@gmail.com>; linan@immunol.org <linan@immunol.org>; yuyz <yuyz@immunol.org>
主　题：Please confirm the authorship of our manuscript "microRNA-199a-3p inhibits hepatic apoptosis and hepatocarcinogenesis by targeting PDCD4"

Dear all co-authors:

For our manuscript entitled "microRNA-199a-3p inhibits hepatic apoptosis and hepatocarcinogenesis by targeting PDCD4", which is submitted to *Oncogenesis* (ONCSIS-20-0261RR), this manuscript has been accepted by *Oncogenesis* currently. This email was to confirm the authorship of this manuscript.

The listed authors are: "Zhenyang Li, Ye Zhou, Liyuan Zhang, Kaiwei Jia, Suyuan Wang, Mu Wang, Nan Li, Yizhi Yu, Xuetao Cao, and Jin Hou". These authors are all from "National Key Laboratory of Medical Immunology & Institute of Immunology, Second Military Medical University, Shanghai 200433, China". The authors "Zhenyang Li, Ye Zhou, Liyuan Zhang" are co-first authors. The authors "Xuetao Cao (caoxt@immunol.org), and Jin Hou (houjin@immunol.org) are co-correspondence.

Please confirm the authorship listed above, and reply this email. Thank you very much for your cooperation.

Yours sincerely,

Jin Hou, Ph.D.
Professor, Institute of Immunology
National Key Laboratory of Medical Immunology
Second Military Medical University
800 Xiangyin Road, Shanghai 200433, China
Phone: (+86 21) 5562 0605; Fax: (+86 21) 6538 2502
Email: houjin@immunol.org

8, Confirmation from Yizhi Yu

邮箱助手 houjin

yuyz 查看相关会话 昨天23:01

收件人: houjin

回复: Please confirm the authorship of our manuscript "microRNA-199a-3p inhibits hepatic apoptosis and hepatocarcinogenesis by targeting PDCD4"

I have confirmed the authorship list which is okay.

Yizhi Yu
Professor, Institute of Immunology
National Key Laboratory of Medical Immunology
Second Military Medical University
800 Xiangyin Road, Shanghai 200433, China
Phone: (+86 21) 5562 0605; Fax: (+86 21) 6538 2502
Email: yuyz@immunol.org

——— 隐藏引用的内容 ———

发件人: houjin <houjin@immunol.org>
发送时间: 2020年9月28日(星期一) 18:41
收件人: houjin <houjin@immunol.org>; caoxt@immunol.org <caoxt@immunol.org>; lzy910722 <lzy910722@126.com>; yzhou1989 <yzhou1989@126.com>; zhangliyuanapril <zhangliyuanapril@163.com>; 15721570382 <15721570382@163.com>; 15021581996 <15021581996@163.com>; wangmukl <wangmukl@gmail.com>; linan@immunol.org <linan@immunol.org>; yuyz <yuyz@immunol.org>
主　题: Please confirm the authorship of our manuscript "microRNA-199a-3p inhibits hepatic apoptosis and hepatocarcinogenesis by targeting PDCD4"

Dear all co-authors:

For our manuscript entitled "microRNA-199a-3p inhibits hepatic apoptosis and hepatocarcinogenesis by targeting PDCD4", which is submitted to *Oncogenesis* (ONCSIS-20-0261RR), this manuscript has been accepted by *Oncogenesis* currently. This email was to confirm the authorship of this manuscript.

The listed authors are: "Zhenyang Li, Ye Zhou, Liyuan Zhang, Kaiwei Jia, Suyuan Wang, Mu Wang, Nan Li, Yizhi Yu, Xuetao Cao, and Jin Hou". These authors are all from "National Key Laboratory of Medical Immunology & Institute of Immunology, Second Military Medical University, Shanghai 200433, China". The authors "Zhenyang Li, Ye Zhou, Liyuan Zhang" are co-first authors. The authors "Xuetao Cao (caoxt@immunol.org), and Jin Hou (houjin@immunol.org) are co-correspondence.

Please confirm the authorship listed above, and reply this email. Thank you very much for your cooperation.

Yours sincerely,

Jin Hou, Ph.D.
Professor, Institute of Immunology
National Key Laboratory of Medical Immunology
Second Military Medical University
800 Xiangyin Road, Shanghai 200433, China
Phone: (+86 21) 5562 0605; Fax: (+86 21) 6538 2502
Email: houjin@immunol.org

9, Confirmation from Xuetao Cao

邮箱助手 houjin

caoxt@immunol.org 查看相关会话 昨天23:30

收件人: houjin lzy910722 yzhou1989 zhangliyuanapril 15721570382 15021581996 wangmukl linan@immunol.org yuyz

Re: Please confirm the authorship of our manuscript "microRNA-199a-3p inhibits hepatic apoptosis and hepatocarcinogenesis by

I confirm the authorship of this manuscript.

Best,

Xuetao Cao

From: houjin
Sent: Monday, September 28, 2020 6:41 PM
To: houjin ; caoxt ; lzy910722 ; yzhou1989 ; zhangliyuanapril ; 15721570382 ; 15021581996 ; wangmukl ; linan ; yuyz
Subject: Please confirm the authorship of our manuscript "microRNA-199a-3p inhibits hepatic apoptosis and hepatocarcinogenesis by targeting PDCD4"

Dear all co-authors:

For our manuscript entitled "microRNA-199a-3p inhibits hepatic apoptosis and hepatocarcinogenesis by targeting PDCD4", which is submitted to *Oncogenesis* (ONCSIS-20-0261RR), this manuscript has been accepted by *Oncogenesis* currently. This email was to confirm the authorship of this manuscript.

The listed authors are: "Zhenyang Li, Ye Zhou, Liyuan Zhang, Kaiwei Jia, Suyuan Wang, Mu Wang, Nan Li, Yizhi Yu, Xuetao Cao, and Jin Hou". These authors are all from "National Key Laboratory of Medical Immunology & Institute of Immunology, Second Military Medical University, Shanghai 200433, China". The authors "Zhenyang Li, Ye Zhou, Liyuan Zhang" are co-first authors. The authors "Xuetao Cao (caoxt@immunol.org), and Jin Hou (houjin@immunol.org) are co-correspondence.

Please confirm the authorship listed above, and reply this email. Thank you very much for your cooperation.

Yours sincerely,

Jin Hou, Ph.D.
Professor, Institute of Immunology
National Key Laboratory of Medical Immunology
Second Military Medical University
800 Xiangyin Road, Shanghai 200433, China
Phone: (+86 21) 5562 0605; Fax: (+86 21) 6538 2502
Email: houjin@immunol.org

10, Confirmation from Myself (Jin Hou)

邮箱助手 houjin

houjin 查看相关会话 18:48

收件人: houjin

回复: Please confirm the authorship of our manuscript "microRNA-199a-3p inhibits hepatic apoptosis and hepatocarcinogenesis by targeting PDCD4"

OK, I confirm that the authorship is right.

Jin Hou.
Institute of Immunology
National Key Laboratory of Medical Immunology
Second Military Medical University

——— 隐藏引用的内容 ———

发件人: houjin <houjin@immunol.org>
发送时间: 2020年9月28日(星期一) 18:41
收件人: houjin <houjin@immunol.org>; caoxt@immunol.org <caoxt@immunol.org>; lzy910722 <lzy910722@126.com>; yzhou1989 <yzhou1989@126.com>; zhangliyuanapril <zhangliyuanapril@163.com>; 15721570382 <15721570382@163.com>; 15021581996 <15021581996@163.com>; wangmukl <wangmukl@gmail.com>; linan@immunol.org <linan@immunol.org>; yuyz <yuyz@immunol.org>
主　题: Please confirm the authorship of our manuscript "microRNA-199a-3p inhibits hepatic apoptosis and hepatocarcinogenesis by targeting PDCD4"

Dear all co-authors:

For our manuscript entitled "microRNA-199a-3p inhibits hepatic apoptosis and hepatocarcinogenesis by targeting PDCD4", which is submitted to *Oncogenesis* (ONCSIS-20-0261RR), this manuscript has been accepted by *Oncogenesis* currently. This email was to confirm the authorship of this manuscript.

The listed authors are: "Zhenyang Li, Ye Zhou, Liyuan Zhang, Kaiwei Jia, Suyuan Wang, Mu Wang, Nan Li, Yizhi Yu, Xuetao Cao, and Jin Hou". These authors are all from "National Key Laboratory of Medical Immunology & Institute of Immunology, Second Military Medical University, Shanghai 200433, China". The authors "Zhenyang Li, Ye Zhou, Liyuan Zhang" are co-first authors. The authors "Xuetao Cao (caoxt@immunol.org), and Jin Hou (houjin@immunol.org) are co-correspondence.

Please confirm the authorship listed above, and reply this email. Thank you very much for your cooperation.

Yours sincerely,

Jin Hou, Ph.D.
Professor, Institute of Immunology
National Key Laboratory of Medical Immunology
Second Military Medical University
800 Xiangyin Road, Shanghai 200433, China
Phone: (+86 21) 5562 0605; Fax: (+86 21) 6538 2502
Email: houjin@immunol.org
