# Supplementary material for: microRNA-199a-3p inhibits hepatic apoptosis and hepatocarcinogenesis by targeting PDCD4
Source: Oncogenesis. 2020 Oct 24;9(10):95. doi: 10.1038/s41389-020-00282-y (PMC7585580; doi:10.1038/s41389-020-00282-y)
Supplement: Supplementary file 2 — Supplementary Information [file 41389_2020_282_MOESM2_ESM.docx]

**microRNA-199a-3p inhibits hepatic apoptosis and hepatocarcinogenesis by targeting PDCD4**

Zhenyang Li, Ye Zhou, Liyuan Zhang, Kaiwei Jia, Suyuan Wang, Mu Wang, Nan Li, Yizhi Yu, Xuetao Cao, and Jin Hou

**Supplementary Figure legends**

Supplementary Fig. S1. Construction of genetic and disease mouse models.

**A,** The *miR*-*199a*-*1* knockout mice were constructed as indicated. **B,** The *miR*-*199a*‑*2* knockout mice were constructed as indicated. **C,** The hepatocyte-specific *miR*-*199a*-*2* knockout mice were constructed and crossed as indicated. **D,** Serum ALT, AST, and GGT were examined in 24-week-old *miR-199a-2^f/f^* and *miR-199a-2^hep-/-^* mice (n=4). **E,** HE staining of liver sections from 24-week-old *miR-199a-2^f/f^* and *miR-199a-2^hep-/-^* mice. **F,** The DEN and DEN plus CCl_4_‑induced hepatocarcinogenesis models were conducted as indicated. Data are shown as mean ± SD or typical photographs of one representative experiment. Similar results were obtained in three independent experiments. ▲P>0.05. Scale bar, 100 μm.

Supplementary Fig. S2. Compensatory hepatocyte proliferation post DEN or APAP injection in *miR*-*199a*‑*2^hep-/-^* mice.

Eight-week-old *miR*-*199a*-*2^f/f^* and *miR*-*199a*-*2^hep-/-^* male mice were treated with DEN (**A**) or APAP (**B**) for the indicated time points. Ki-67 staining for cell proliferation was examined in liver sections. Data are shown as typical photographs of one representative experiment. Similar results were obtained in three independent experiments. Scale bars, 100 μm.

Supplementary Fig. S3 Hepatocyte-specific miR-199a-3p knockout promotes apoptosis through the mitochondrial pathway.

(**A, B**), BNL CL.2 (**A**) and HL-7702 (**B**) cells were treated with APAP or TNF-α (50 ng/ml) plus CHX (25 µg/ml) for 24 hours as indicated, and cleaved caspase-8 were detected by Western blot. (**C, D**), Mice were treated with APAP (**C**), DEN (**D**), or LPS (40 µg/kg) plus D-gal (300 mg/kg) for 6 hours as indicated, and cleaved caspase-8 were detected by Western blot. (**E, F**), Control and *miR‑199a-2^-/-^* cell lines were stimulated by APAP and the indicated proteins were examined by Western blot. (**G, H**), *miR‑199a‑2^f/f^* and *miR‑199a-2^hep-/-^* mice were administrated with APAP (**G**) or DEN (**H**) for the indicated time periods, the indicated proteins were examined by Western blot. (**I-L**), The release of cytochrome C was examined in the cytoplasmic proteins excluding mitochondria from cells or liver tissues upon APAP or DEN administration as indicated. Data are shown as typical photographs of one representative experiment. Similar results were obtained in three independent experiments.

Supplementary Fig. S4. miR-199a-3p targets PDCD4 at post-transcriptional level to inhibit hepatocyte apoptosis

**A,** Eight consistent increased proteins (>1.5 fold) in the liver of *miR*-*199a*-*2^hep-/-^* vs *miR*‑*199a*-*2^f/f^* were listed. **B,** Sequence alignment of miR-199a-3p and its putative conserved target site in *pdcd4* mRNA among the indicated species. (**C, D),** The mRNA level of *pdcd4* was detected in the liver tissues and primary hepatocytes from *miR*‑*199a*‑*2^f/f^* or *miR*-*199a*-*2^hep-/-^* mice (**C**), and in control or *miR*-*199a*-*2* knockout hepatocyte cell lines (**D**). **E,** PDCD4 and cleaved caspase-3 were detected by Western blot in PDCD4-overexpressed hepatocyte cell lines treated with APAP for the indicated time points. Data are shown as mean ± SD or typical photographs of one representative experiment. Similar results were obtained in three independent experiments. ▲P>0.05.

Supplementary Fig. S5. miR-199a-3p-suppressed hepatocyte apoptosis is dependent on PDCD4.

**A,** miR-199a-3p mimics were transfected into control or *pdcd4* knockout hepatocyte cell lines. PDCD4 and cleaved caspase-3 were measured by Western blot in the indicated time points post APAP treatment. **B,** PDCD4 was overexpressed in control or *miR*-*199a*-*2* knockout hepatocyte cell lines. PDCD4 and cleaved caspase-3 were measured by Western blot in the indicated time points post APAP treatment. Data are shown as typical photographs of one representative experiment. Similar results were obtained in three independent experiments.
