# Supplementary material for: microRNA-199a-3p inhibits hepatic apoptosis and hepatocarcinogenesis by targeting PDCD4
Source: Oncogenesis. 2020 Oct 24;9(10):95. doi: 10.1038/s41389-020-00282-y (PMC7585580; doi:10.1038/s41389-020-00282-y)
Supplement: Supplementary file 3 — Supplementary tables [file 41389_2020_282_MOESM3_ESM.docx]

**Supplementary Tables**

Supplementary Table 1. Primer sequences for identification of mice.

| *miR-199a-1*  *miR-199a-2*  *miR-199a-2 flox*  *Alb-cre* | Forward  Reverse  Forward  Reverse  Forward  Reverse  Common  Wildtype Forward  Mutant Forward | TCCAGATGTGAGCAAGTGGC  CCAAGATAAAGACCAGCAGG  GGCGATTCTAGCGGTCTCTC  TCACGGTTATCTGTCCCTTAGC  CAAAGTATCGCAGGAAAGTTAA  TACCAGATGTTTTCGTGTAATG  TTGGCCCCTTACCATAACTG  TGCAAACATCACATGCACAC  GAAGCAGAAGCTTAGGAAGATGG |
| --- | --- | --- |

Supplementary Table 2. gRNA sequences for construction of knockout cell

| HL-7702 *miR-199a-2* KO  BNL CL.2 *miR‑199a‑2* KO  HL-7702 *pdcd4* KO  BNL CL.2 *pdcd4* KO | gRNA-1 sense  gRNA-1 anti-sense  gRNA-2 sense  gRNA-2 anti-sense  gRNA-1 sense  gRNA-1 anti-sense  gRNA-2 sense  gRNA-2 anti-sense  gRNA-1 sense  gRNA-1 anti-sense  gRNA-2 sense  gRNA-2 anti-sense  gRNA-1 sense  gRNA-1 anti-sense  gRNA-2 sense  gRNA-2 anti-sense | GAGCAACGCCATGGACCGCT  AGCGGTCCATGGCGTTGCTC  TAGTCTGAACACTGGGGCGA  TCGCCCCAGTGTTCAGACTA  GAACACTGGGGCGACGGAGC  GCTCCGTCGCCCCAGTGTTC  CCAGCCGTCCATGGCGTTGC  GCAACGCCATGGACGGCTGG  ATAACATATTGCTAGTTGTA  TACAACTAGCAATATGTTAT  ATTTACTCTCCGTCTTCTAT  ATAGAAGACGGAGAGTAAAT  TAGCATGATGGTGGTCAATA  TATTGACCACCATCATGCTA  TAGCCTGGGCTGCCGGTATC  GATACCGGCAGCCCAGGCTA |
| --- | --- | --- |

Supplementary Table 3. RT primer sequences and real-time quantitative PCR primer sequences

| miR-199a-3p RT  miR-199a-5p RT  U6  U6  miR-199a-3p  miR-199a-5p  miRNA Universal  mouse β-actin  mouse β-actin  mouse pdcd4  mouse pdcd4  human pdcd4  human pdcd4 | Forward  Reverse  Forward  Forward  Reverse  Forward  Reverse  Forward  Reverse  Forward  Reverse | GTCGTATCCAGTGCAGGGTCCGAGGT  ATTCGCACTGGATACGACTAACCA  GTCGTATCCAGTGCAGGGTCCGAGGTA  TTCGCACTGGATACGACGAACAG  CTCGCTTCGGCAGCACA  AACGCTTCACGAATTTGCGT  GTCACAGTAGTCTGCACAT  CCCAGTGTTCAGACTACC  GTGCAGGGTCCGAGGT  AGTGTGACGTTGACATCCGT  GCAGCTCAGTAACAGTCCGC  AAAGACGACTGCGGAAAAATTCA  CTTCTAACCGCTTCACTTCCATT  GCAAAAAGGCGACTAAGGAAAAA  TAAGGGCGTCACTCCCACT |  |  |
| --- | --- | --- | --- | --- |
