# Supplementary figures and images for: microRNA-199a-3p inhibits hepatic apoptosis and hepatocarcinogenesis by targeting PDCD4
Source: Oncogenesis. 2020 Oct 24;9(10):95. doi: 10.1038/s41389-020-00282-y (PMC7585580; doi:10.1038/s41389-020-00282-y)

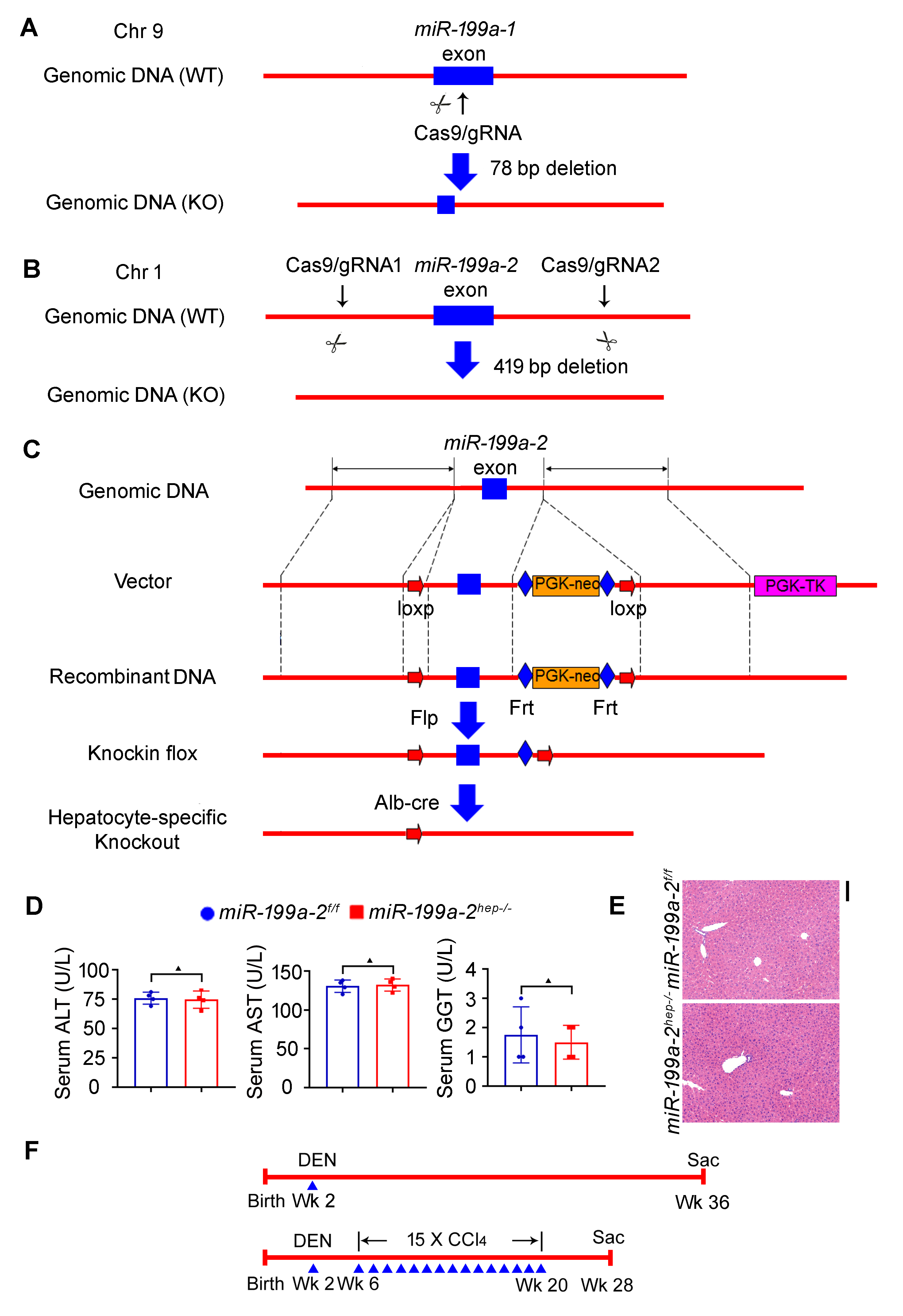

Supplement: Supplementary file 4 — Figure S1 [file 41389_2020_282_MOESM4_ESM.tif]

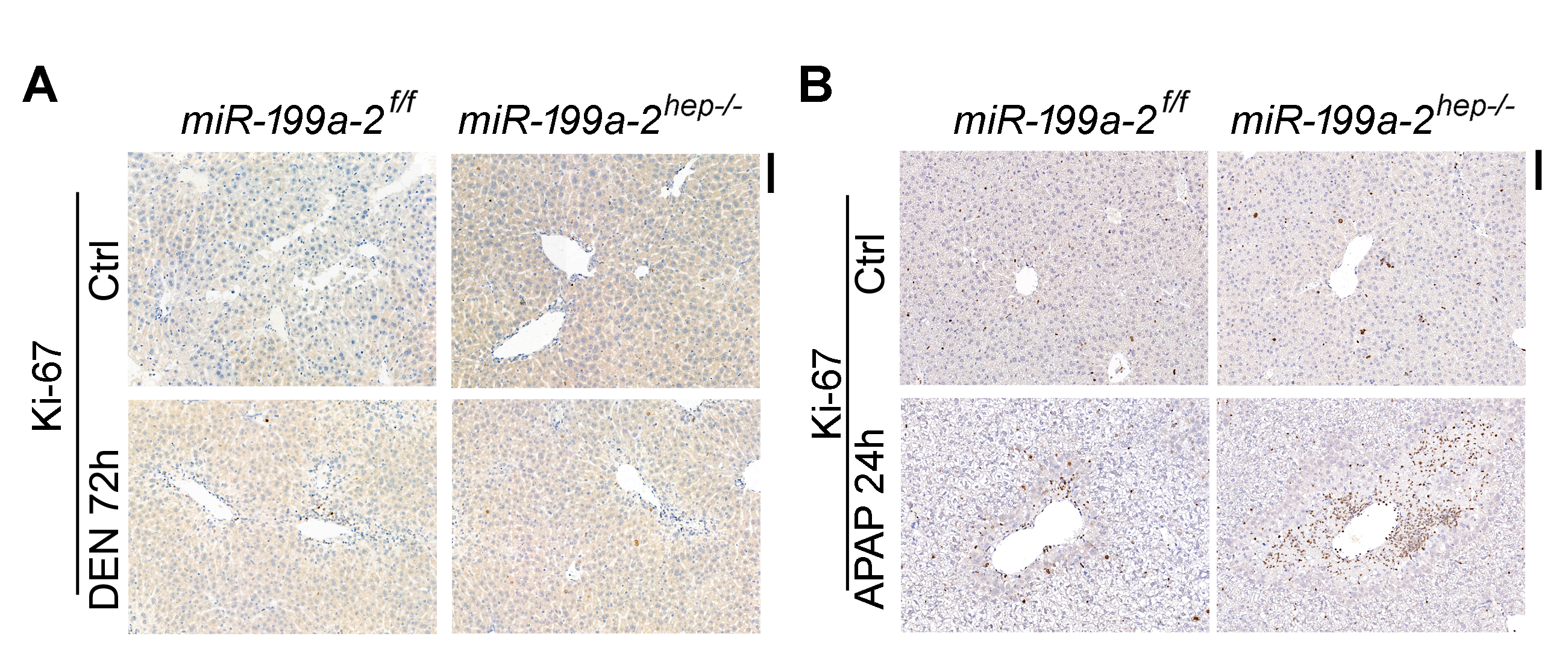

Supplement: Supplementary file 5 — Figure S2 [file 41389_2020_282_MOESM5_ESM.tif]

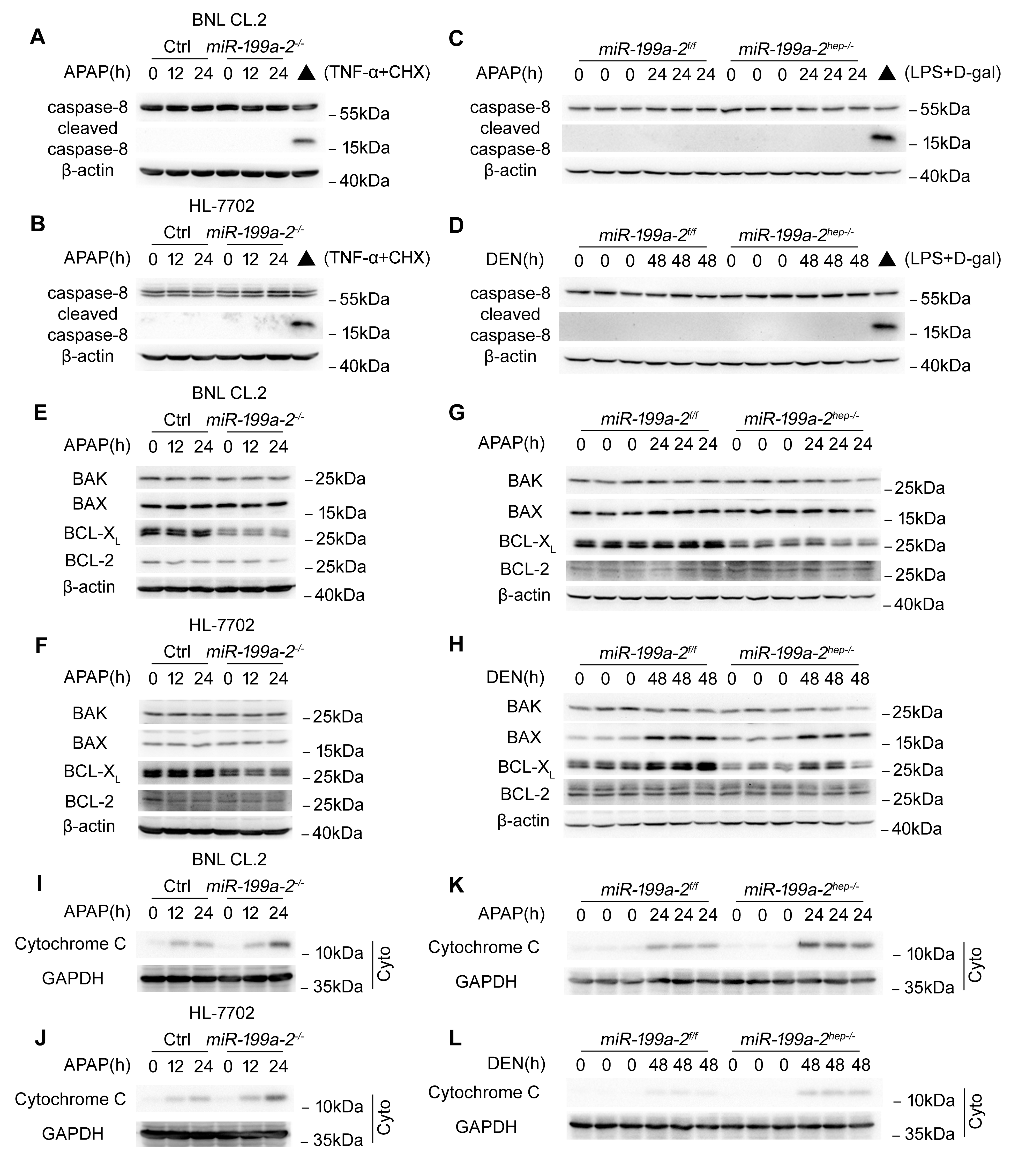

Supplement: Supplementary file 6 — Figure S3 [file 41389_2020_282_MOESM6_ESM.tif]

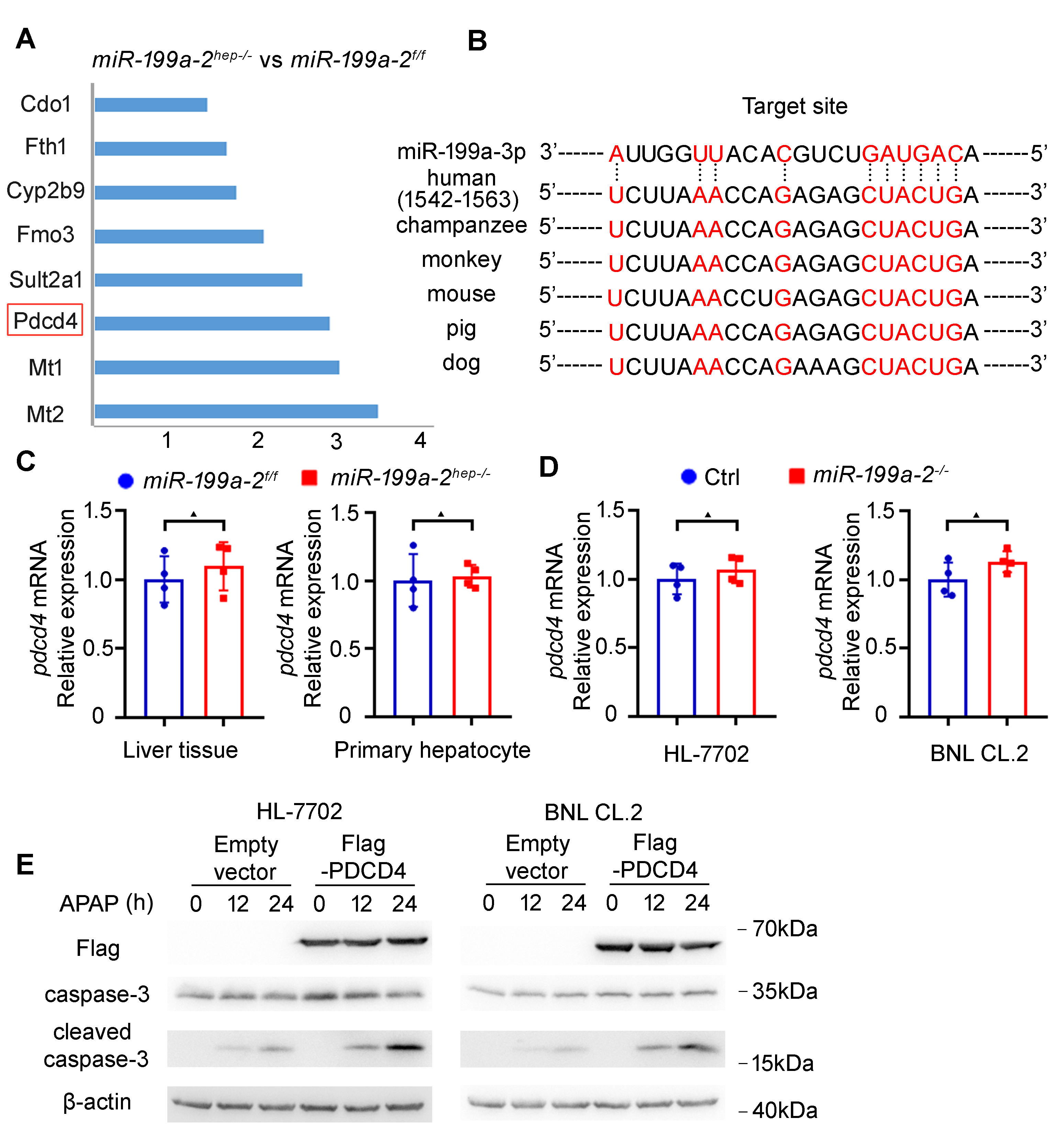

Supplement: Supplementary file 7 — Figure S4 [file 41389_2020_282_MOESM7_ESM.tif]

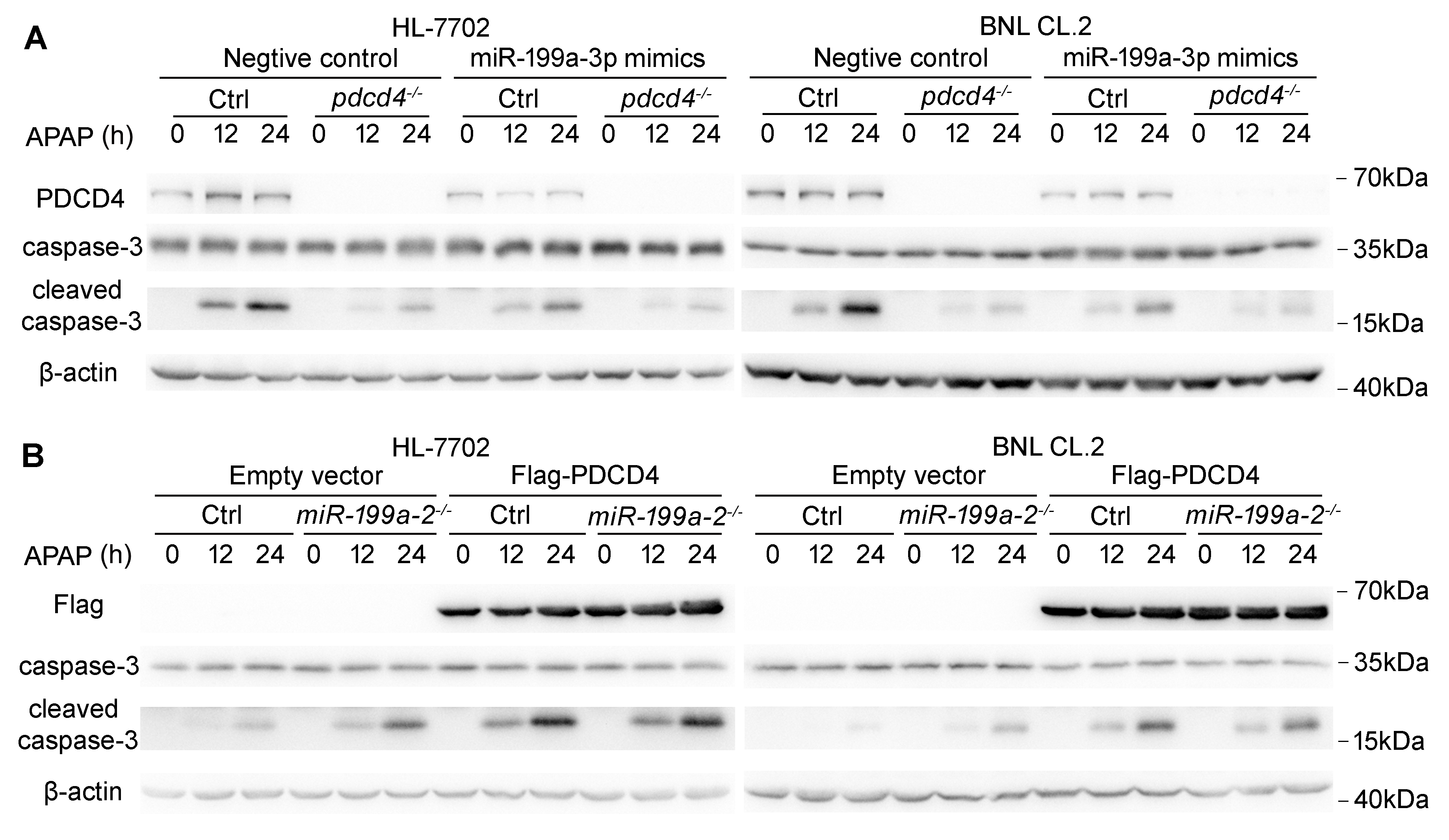

Supplement: Supplementary file 8 — Figure S5 [file 41389_2020_282_MOESM8_ESM.tif]
